# Transcriptome sequencing and weighted correlation network analysis reveal the molecular mechanism of growth variation in highly resistant transgenic double *Bt* poplar

**DOI:** 10.1186/s12870-026-08431-y

**Published:** 2026-02-26

**Authors:** Ziyan He, Huan Zhang, Lingyun Liu, Yang Liu, Zihui Liu, Jinmao Wang, Yachao Ren, Minsheng Yang

**Affiliations:** 1 Institute of Forest Biotechnology, Forestry College, Hebei Agricultural University, Baoding, 071000 China; 2 Hebei Key Laboratory for Tree Genetic Resources and Forest Protection, Baoding, 071000 China

**Keywords:** *Bt* gene, Poplar, Growth variation, Differentially expressed genes, Weighted gene co-expression network analysis (WGCNA), Hub genes

## Abstract

The *Bt* gene is widely used in insect-resistant transgenic plants, but its integration (especially dual *Bt* genes) may cause unintended growth effects. To explore the molecular mechanism of *Bt* genes regulating the growth of recipient plants, we used transgenic single *Bt* gene poplar 741, transgenic double *Bt* gene poplar 741 obtained through secondary transformation, and wild-type (741) as research subjects, combined with physiological determination and transcriptome sequencing. The results showed that, compared to 741 and the single *Bt* lines, double *Bt* lines exhibited noticeable variations, including reduced plant height and slow growth. Transcriptome analysis revealed that the numbers of differentially expressed genes (DEGs) in the three dual *Bt* transgenic lines (pc2, pc3, and pc9) relative to the wild-type were 1126, 920, and 1224, respectively. These values were notably higher than those detected in the transgenic single *Bt* lines pb29 (Integrated *Cry1Ac*) and CC84 (Integrated *Cry3A*), which exhibited 726 and 112 DEGs, respectively. More DEGs were observed in transgenic double *Bt* gene poplar 741 compared to 741 and CC84; the number of upregulated genes exceeded the number of downregulated genes. Fewer DEGs were identified relative to the pb29. The DEGs were mainly involved in metabolic processes such as starch and sucrose metabolism, pentose and glucuronate interconversions, plant hormone signal transduction, and flavonoid biosynthesis. Weighted gene co-expression network analysis (WGCNA) identified blue and brown modules significantly correlated with growth traits, and screened hub genes including CBL - interacting serine/threonine - protein kinase 11 and protein disulfide-isomerase A1 among others. These results indicated that the high expression and interaction of dual *Bt* genes disrupted the expression of growth - related genes in multiple metabolic pathways, such as plant hormone signal transduction, phenylpropanoid biosynthesis, and flavonoid biosynthesis, leading to metabolic disorders and phenotypic variation of poplar 741.

**Supplementary Information:**

The online version contains supplementary material available at 10.1186/s12870-026-08431-y.

## Introduction

The Poplar 741 (*Populus × aldatomentosa* CL 741) is an elite clone of the *Populus* section *Leuce* and serves as a key species for artificial forests in China. However, as the area of artificial forests continues to expand, challenges posed by poplar diseases and pests are becoming increasingly serious. Genetic engineering methods, which introduce insect resistance genes into the poplar genome, can partially enhance its pest resistance. This provides opportunities for the developing new insect-resistant poplar varieties [1–3]. In 1991, Wu [4] successfully transferred the *Bt* gene to European black poplar (*Populas nigra*) for the first time; this was the prelude to insect resistance transgenic research. In 1993, Tian transformed a specific *Bt* gene that is toxic to Lepidopteran insects into European black poplar [5]. However, single resistance genes are prone to driving adaptive evolution in pests, and their potential unintended effects on tree growth remain insufficiently understood, which limits large-scale adoption and long-term safety. One research team adopted a secondary transformation method to introduce the *BtCry3A* gene into transgenic *BtCry1Ac* gene poplar 741, thereby obtaining transgenic poplar 741 lines with both *Cry3A* and *Cry1Ac* genes [6]. Further studies on these transgenic lines confirmed the expression of *Cry1Ac* and *Cry3A* insecticidal proteins. These proteins resulted in high mortality rates against larvae of both the *Coleoptera Plagiodera versicolora* and the *Lepidoptera Hyphantria cunea* [7]. The combined use of multiple insect-resistance genes not only broadens the spectrum of insect resistance but also effectively utilizes the gene stacking effect [8]. The stacking of *Cry1Ac* and *Cry1Ig* will not only enhance protection against insect attack but also delay the development of resistance to *Bt* toxins [9]. However, after planting and cultivating the transgenic double *Bt* gene poplar 741 lines in an experimental field, researchers observed notable differences compared with the wild type. The transgenic lines showed slower growth, reduced plant height, weaker vigor, withered shoots, and even gradual death in some cases. The underlying causes of these effects remain unclear.

The integration and expression of exogenous genes can enhance the target traits of recipient plants, but they may also induce unintended effects. Phenotypic variations have been reported in transgenic Arabidopsis (*Arabidopsis thaliana*), rice (*Oryza sativa*), and tomato (*Solanum lycopersicum*). These variations include plant dwarfism, excessive height, wrinkled or deformed leaf shapes, and changes in leaf color [10–13] These variations may result from two main factors. First, overexpression or RNA interference caused by exogenous genes can lead to gene silencing. Second, T-DNA insertion may activate or inactivate other endogenous genes [14, 15]. The introduction of exogenous *Bt* genes into the recipient plant genome may have adverse effects on internal physiological metabolism [16, 17] or alter its agronomic traits [18–21]. Bai [22] found that the upregulated expression of two key m6A methyltransferase genes, *PxMettl3* and *PxMettl14*, in *Plutella xylostella* could promote the increase of m6A methylation level of *PxJHE* gene, thereby inhibiting the expression of *PxJHE* gene and inducing a significant increase of Juvenile hormone content in *Plutella xylostella*, enabling it to develop high resistance to *BtCry1Ac* insecticidal protein while maintaining normal growth and development. Substantial differences in plant height, biomass, chlorophyll content, and other nutritional growth indicators have been observed between the transgenic insect-resistant rice Huahui-1 and its parent variety Minghui-63 under both agricultural and saline-alkali growth conditions [23]. However, the molecular mechanisms responsible for these phenotypic differences have not yet been determined. In a comparative growth study of transgenic *Bt* gene poplar 741 and the wild type, the pb29 transgenic *Cry1Ac* gene poplar 741 line showed smaller plant height and diameter at breast height than both the wild type and the transgenic *Cry3A* gene poplar 741 line CC84. Transcriptome analysis has preliminarily identified the differential expression of key genes in plant hormone signaling pathways [24]. However, the molecular mechanisms underlying these unintended effects remain unclear.

To clarify the growth differences between transgenic double *Bt* gene poplar 741 and the wild type, and to explore the causes of phenotypic variation in the transgenic double *Bt* gene poplar 741, we used transgenic single and double *Bt* gene poplar 741 lines and the wild type as experimental materials. We measured growth and physiological indicators in potted seedlings that exhibited significant differences in growth. Through transcriptome sequencing and weighted gene co-expression network analysis (WGCNA), candidate genes and metabolic pathways associated with trait variation in the transgenic double *Bt* gene poplar 741 were identified. This study aimed to uncover the molecular mechanisms underlying the unintended effects caused by the integration and expression of the double *Bt* genes in poplar 741, thus providing valuable references for the exploration of unintended effects in transgenic *Bt* plants.

## Materials and methods

### Experimental materials

The experimental material used in this study included the transgenic *Cry1Ac* gene poplar 741 line pb29, which exhibits high resistance to Lepidoptera pests such as *Hyphantria cunea*. The transgenic *Cry3A* gene poplar 741 line CC84 shows high resistance to beetles such as *Plagiodera versicolora*. The transgenic double *Bt* gene poplar 741 lines pc2, pc3, and pc9, obtained through secondary transformation (using pb29 as the recipient material and transferring the *Cry3A* gene), exhibit high resistance to both Lepidoptera and Coleoptera pests. The wild type was poplar 741. The types and sources of experimental materials are shown in Table S1 [25–28]. All types of poplar tissue culture seedlings are preserved by the Forest Genetics and Breeding Laboratory of Hebei Agricultural University. Exogenous gene expression patterns and insect resistance effects for each line have been reported by Wang Guiying [29] (Table S2). The growth performance of the secondary-transformed double *Bt* gene poplar 741 (planted in the experimental field by our research group) is shown in Fig. S1, along with the performances of the wild type and single *Bt* gene poplar 741. PCR detection of the double *Bt* gene is shown in Fig. S2, and the primers used are listed in Table S3.

### Experimental design

The experiment was conducted using tissue-cultured seedlings of transgenic single and double *Bt* gene 741 poplars, as well as wild-type seedlings, all selected for robust growth and uniform vigor. The seedlings were transplanted into plastic pots (inner diameter: 15.5 cm; bottom diameter: 10 cm; height: 11 cm) filled with a nursery substrate consisting of field soil, nutrient soil, and perlite in a 3:2:1 ratio. One seedling was planted in each pot (15 replicates per line). The pots were placed in an artificial climate chamber for cultivation. In the chamber, the daytime temperature was set to 27–30 °C, whereas the nighttime temperature was maintained at 20–22 °C. The relative humidity was kept at approximately 75%. A white light source was used, with an illumination intensity of 8000 lx and photoperiod of 14 h·d⁻¹. After 60 days of cultivation in the artificial climate chamber (i.e., until significant growth differences were observed among the lines), the relevant indicators were measured.

### Determination of phenotype-related indicators of transgenic double *Bt* gene poplar 741

#### Determination of growth-related indicators

Three plants were randomly selected from each line. Plant height and internode spacing were measured and recorded using a standard ruler (accuracy: 0.1 cm). Ground diameter was measured using a vernier caliper (accuracy: 0.001 cm). The number of leaves per plant was counted, and the third fully expanded leaf was selected. Leaf length and width were measured using a standard ruler (accuracy: 0.1 cm). Three replicates were performed for each line. Additionally, three intact plants were randomly selected from each line and separated into roots, stems, and leaves, which were placed in envelopes and dried in an oven. Their dry weights were measured, and three replicates were conducted for each line. The specific procedures followed the method described by Ren et al. [30].

#### Determination of photosynthetic pigment content in leaves

The third functional leaves of plants from each line were collected. After removal of the midribs, 0.1 g of fresh leaf tissue was cut into pieces and soaked in 10 mL of 95% ethanol for pigment extraction. The specific procedures and methods for determining pigment content followed the protocols described by Liu Xijun et al. [31] and Ren et al. [38]. Three replicates were performed for each line.

#### Determination of photosynthesis parameters and chlorophyll fluorescence parameters

The third functional leaves of each line were selected. Photosynthetic parameters, including net photosynthetic rate (*Pn*), stomatal conductance (*Cond*), intercellular CO₂ concentration (*Ci*), and transpiration rate (*Tr*), were measured using an LI-6400XT portable photosynthesis system (LI-COR, USA). Chlorophyll fluorescence parameters were determined in vivo using a Handy PEA plant efficiency analyzer (PEA Plus Version 1.00, Hansatech, UK). The main parameters measured were the maximum photochemical efficiency of PSII (*Fv/Fm*), potential activity of PSII (*Fv/Fo*), and photosynthetic performance index (*PI*) after 20 min of dark adaptation. Each line was measured with three replicates.

#### Determination of plant hormone content

The 3rd to 4th functional leaves of each line were collected, and five plant hormones—indole-3-acetic acid (IAA), indole-3-butyric acid (IBA), gibberellin (GA), cytokinin (CTK), and abscisic acid (ABA)—were quantified using an enzyme-linked immunosorbent assay kit (Enzyme-linked Biology, Shanghai) following the manufacturer’s instructions. Each line was analyzed in triplicate.

### RNA sequencing

Three plants were randomly selected from each line, and the third functional leaf was harvested and frozen in liquid nitrogen. Total RNA was extracted using a polysaccharide polyphenol plant total RNA extraction kit (Tiangen Biochemical Technology, Beijing), in accordance with the manufacturer’s instructions. RNA purity (A_260/280_ ratio) was assessed using a NanoDrop ND-1000 spectrophotometer (NanoDrop, USA); RNA concentration and integrity were evaluated using an Agilent 2100 Bioanalyzer (Agilent Technologies, USA). A sequencing company was commissioned to construct cDNA libraries and perform high-throughput sequencing for each sample using the Illumina HiSeq™ 2000 platform (Illumina, USA) with paired-end 150 bp sequencing.

#### Sequence alignment and differential expression analysis

Clean reads were aligned to the reference genome of *Populus trichocarpa* (Populus_trichocarpa.JGI2.0.30.dna_sm.toplevel.fa) using STAR software (v2.5) [32]. The alignment results for each sample were statistically analyzed. Gene annotation was performed using the reference genome annotation file (Populus_trichocarpa.JGI2.0.35.gtf.gz). Genome-wide coverage and RNA expression were calculated based on comparisons with the reference genome. RNA expression levels were normalized via Cufflinks software (v2.2.1) [33], using the transcripts per million mapped reads method [34]. Differentially expressed genes (DEGs) were identified using the edgeR package in R [35]. The fold change (FC) between the two groups was calculated using the formula: logFC = log_2_(treatment group/control group). Genes with |logFC > 1 and *q*-value < 0.05 were considered DEGs. Three sets of threshold combinations were set for sensitivity analysis, all using q-value to control statistical significance while accommodating different fold-change filtering requirements: |logFC| > 0.58 (≥ 1.5-fold change) with *q* < 0.05, |logFC| > 1 with *q* < 0.01, and |logFC| > 2 with *q* < 0.05. The numbers of upregulated and downregulated genes were determined based on logFC values. All DEGs were subjected to expression pattern clustering analysis using K-means hierarchical clustering software.

#### DEG functional enrichment analysis and transcription factor prediction

Functional enrichment and metabolic pathway analyses of the DEGs were performed using the Gene Ontology (GO) and Kyoto Encyclopedia of Genes and Genomes (KEGG) databases. For GO terms, a *q*-value < 0.05 was regarded as the threshold for significantly enriched DEGs; for KEGG pathway enrichment analysis, a *p*-value < 0.05 served as the significance threshold. Transcription factors in each comparison group were identified using the Plant Transcription Factor Database (PlantTFDB), and their expression patterns were subsequently analyzed.

### Quantitative Reverse Transcription Polymerase Chain Reaction (RT-qPCR) validation

Ten DEGs were randomly selected to verify the reliability of the transcriptome sequencing results via RT-qPCR, using actin (GenBank accession number: EF418792.1) as the internal reference gene. Based on sequence information for each gene, primers were designed using Primer Premier 6.0 software. Primer details are provided in Supplementary Table S4. RNA samples obtained from transcriptome sequencing were reverse-transcribed into cDNA using the HisScript II Q RT SuperMix for qPCR (+ gDNA wiper) reverse transcription kit (Vazyme Biotech, Nanjing). RT-qPCR was performed using the MagicSYBR mixture (Kangwei Century, Taizhou), a fluorescent quantitative mix, and the Stratagene Mx3005P Real-Time PCR system (Agilent Technologies, USA). The PCR reaction system and procedure were established following the manufacturer’s instructions, and three replicates were conducted for each experiment. RT-qPCR results were analyzed using the 2^−ΔΔCT^ method to calculate the relative expression levels of each gene [36].

### Weighted gene co-expression network analysis

The WGCNA package in R software was used to construct a gene co-expression network. After the co-expression gene modules were successfully established, a correlation analysis was performed with 20 trait indicators (see Supplementary Table S5). Pearson correlation was used for the sample-trait correlation analysis. Modules related to the traits of interest were identified based on the correlation between module eigengenes and traits, as well as the associated *p*-values. Gene expression levels in the target modules were then analyzed, and hub genes were screened based on gene connectivity. The gene co-expression network was visualized using Cytoscape software. An in-depth exploration of the hub genes associated with growth regulation in double *Bt* gene poplar 741 was conducted through WGCNA and gene set–phenotype association analysis.

### Statistical analysis

The data were organized and statistically analyzed using Microsoft Excel 2016 and DPS 7.05 software. One-way analysis of variance was performed for each treatment, and Duncan’s new multiple range test was used for significance testing (*p* < 0.05). All data were expressed as the mean ± standard deviation of three replicates. Figures were designed using Microsoft Excel 2016 and OriginPro 2018 software.

## Results and analysis

### Comparative morphological analysis of different types of transgenic poplar 741

**Fig. 1:**
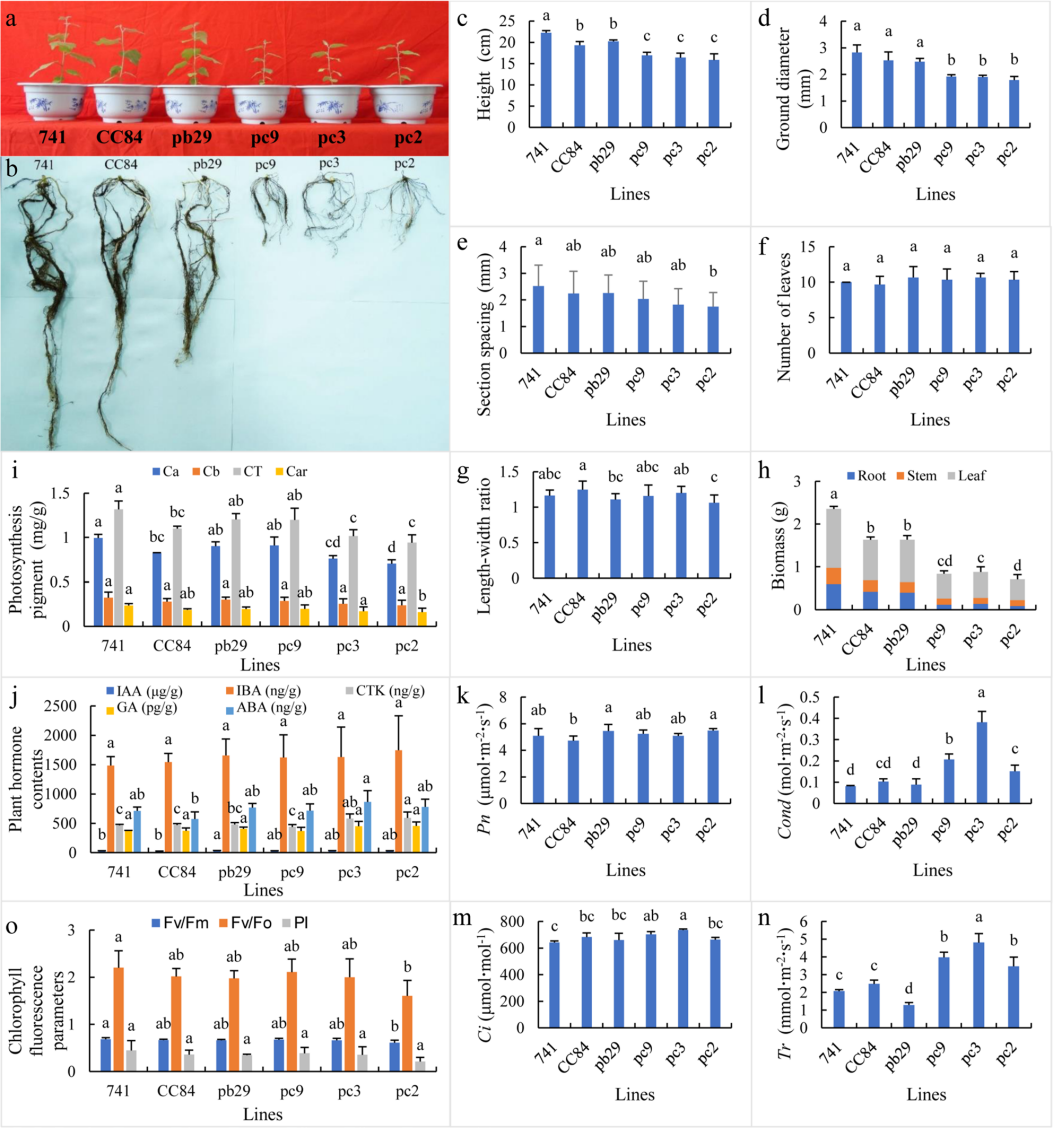
Comparison of potted seedling growth among different types of poplar 741. Note: **a**: Growth status of different types of poplar 741 potted seedlings; **b**: Comparison of root systems of different types of poplar 741 trees; **c**: Heights of different types of poplar 741 trees; **d**: Ground diameters of different types of poplar 741 tree; **e**: Internode spacing of different types of poplar 741; **f**: Leaves of different types of poplar 741 trees; **g**: Leaf length-to-width ratios of different types of poplar 741 trees; **h**: Biomasses of different types of poplar 741 trees; **i**: Comparison of photosynthetic pigment content in different types of poplar 741 leaves; **j**: Comparison of hormone contents in different types of poplar 741 leaves; **k**: Comparison of photosynthetic rates in different types of poplar 741 trees; **l**: Comparison of stomatal conductances in different types of poplar 741 trees; **m**: Comparison of intercellular carbon dioxide concentrations in different types of poplar 741 trees; **n**: Comparison of transpiration rates in different types of poplar 741 trees; and **o**: Comparison of chlorophyll fluorescence parameters in different types of poplar 741 trees. Different lowercase letters in the same column indicate significant differences (*p* < 0.05)

When the growth of transgenic double *Bt* gene poplar 741 showed significant differences from that of the transgenic single *Bt* gene poplar 741 and the wild type (Fig. 1a), growth-related indices were evaluated. After 60 days of pot cultivation, the root systems of the three transgenic double *Bt* gene poplar 741 lines—pc2, pc3, and pc9—were significantly smaller than those of the wild type and the single *Bt* gene transgenic poplar 741 lines pb29 and CC84 (Fig. 1b). Plant height, ground diameter, and biomass were significantly lower in the double *Bt* lines compared to the wild type and single *Bt* gene transgenic lines (Fig. 1c and d, 1 h). Internode length was also shorter than in the wild type and single *Bt* gene lines (Fig. 1e); the number of leaves was higher than in the wild type and CC84 (Fig. 1f). No significant difference was observed in the leaf length-to-width ratio between the transgenic lines and the wild type (Fig. 1g). The chlorophyll a (Ca), chlorophyll b (Cb), total chlorophyll (CT), and carotenoid (Car) contents in single and double *Bt* gene-transformed poplar 741 were lower than those in the wild type (Fig. 1i). The IAA, IBA, and ABA contents in the double *Bt*-transgenic poplar 741 lines and pb29 were higher than those in the wild type and CC84. The CTK and GA contents in pc2, pc3, and pb29 were also higher than in the wild type and CC84 (Fig. 1j). The photosynthetic parameters *Cond* and *Tr* in the double *Bt*-transgenic poplar 741 were significantly increased compared to the wild type and single *Bt*-transgenic poplar 741, whereas no significant difference in *Pn* was detected between the double *Bt*-transgenic poplar 741 and the wild type. No significant differences in *Pn*, *Cond*, or *Ci* were observed between the single *Bt*-transgenic 741 poplar and the wild type (Figs. 1k–n). The *Fv/Fm*, *Fv/Fo*, and *PI* values of both single and double *Bt* gene transgenic poplar 741 were lower than those of the wild type. Notable differences were observed between the transgenic double *Bt* gene poplar 741 and the transgenic single *Bt* gene poplar 741 (Fig. 1o). Overall, compared to the wild type and the transgenic single *Bt* gene poplar 741, significant changes were observed in the photosynthetic pigments, plant hormone levels, photosynthetic parameters, and chlorophyll fluorescence parameters of the transgenic double *Bt* gene poplar 741. Compared to the wild type and single *Bt* gene-transformed poplar 741, the growth of double *Bt* gene-transformed poplar 741 was significantly inhibited, whereas the growth of single *Bt* gene-transformed poplar 741 was moderately inhibited relative to the wild type.

### Analysis of DEGs

#### Identification of DEGs and hierarchical clustering analysis

**Fig. 2:**
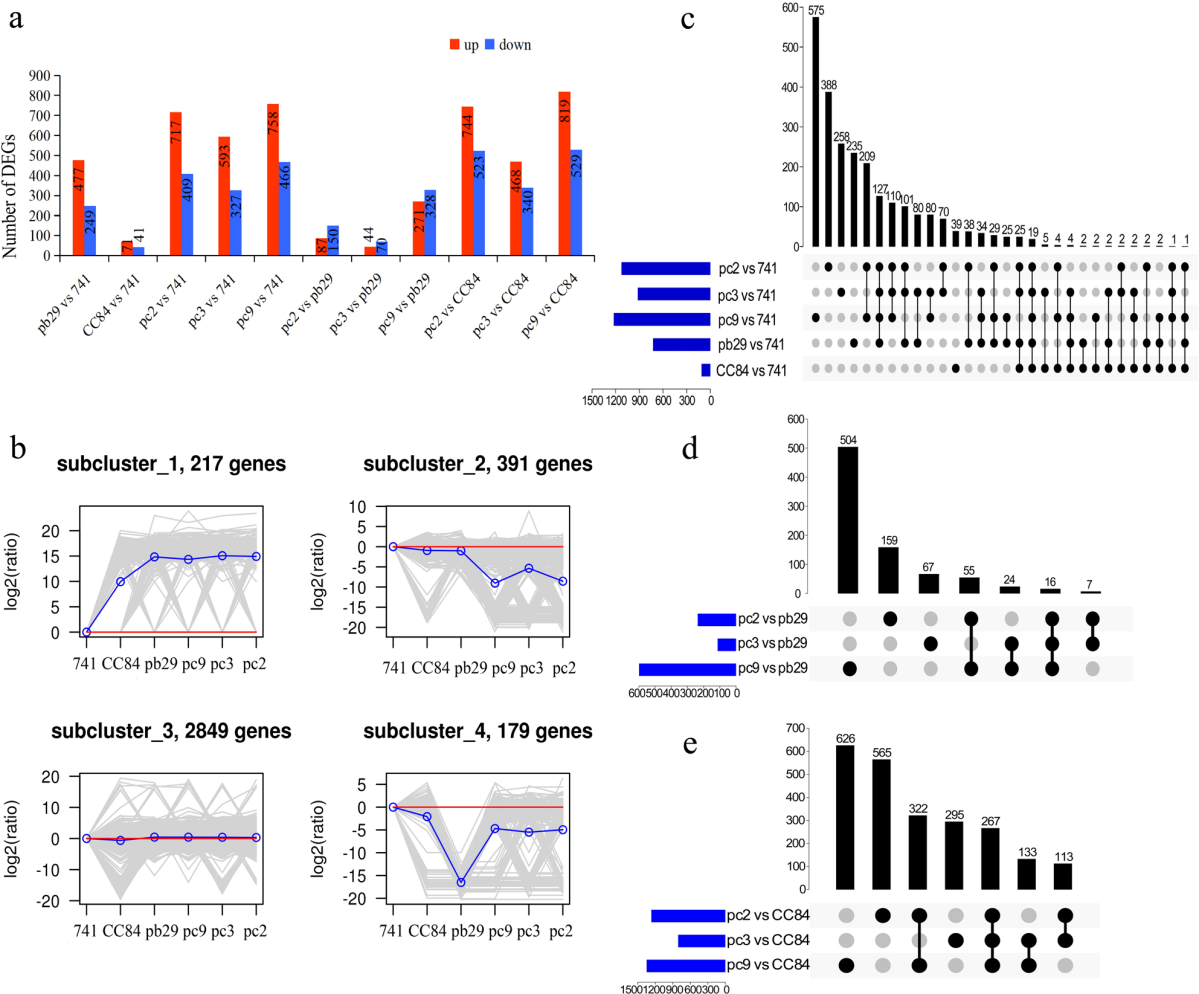
Analysis of DEGs. Note: **a**: Statistics regarding the number of differentially expressed genes (DEGs); **b**: Hierarchical clustering of DEGs; **c**: UpSet plot of five comparison groups of five transgenic lines compared to the wild type; **d**: UpSet plot of three comparison groups of transgenic double Bt gene poplar 741 compared to pb29; and **e**: UpSet plot of three comparison groups of transgenic double Bt gene poplar 741 compared to CC84. Different lowercase letters in the same column indicate significant differences (*p* < 0.05)

Transcriptome sequencing was performed on the leaves of different types of poplar 741 trees, yielding a total of 154.65 Gb of clean data. The guanine-cytosine (GC) content ranged from 43.5% to 45.5%, with Q20 and Q30 values exceeding 92% and 81%, respectively (Table S6). The sequences from each sample were aligned to the reference genome of *P. trichocarpa*, with alignment rates exceeding 84% (Table S6). Statistical analysis of DEGs among different types of poplar 741 is shown in Fig. 2a. Multi-threshold gradient sensitivity analysis (Fig. S3) demonstrated that the core DEG set remained essentially unchanged regardless of adjustments to fold-change or moderate relaxation of significance thresholds. The original threshold (|logFC| > 1, *q* < 0.05) yielded statistically rigorous, biologically meaningful, and reproducible DEGs, with robust and reliable results that did not lead to omission or bias of key biological information. A larger number of DEGs was observed between the double *Bt*-transgenic poplar 741 and either the wild type or CC84, whereas fewer DEGs were identified between the double *Bt*-transgenic poplar 741 and pb29. Additionally, more DEGs were detected between pb29 and the wild type, whereas fewer DEGs were found between CC84 and the wild type. Compared to the wild type, the five transgenic poplar 741 lines exhibited more upregulated genes than downregulated genes. Among the different lines, pc2, pc3, and pc9 had a greater number of DEGs than the single *Bt* gene-transformed lines pb29 and CC84. The heatmap of the top 50 most significantly up- and down-regulated DEGs between pc2, pc3, pc9 and wild-type is shown in Fig. S4. Integrated gene expression analysis identified a core set of significantly differentially expressed genes across transgenic lines pc2, pc3, and pc9. Significantly upregulated genes include those encoding ribulose-phosphate 3-epimerase, cytoplasmic isoform, chlorophyll a-b binding protein 7, chloroplastic, protein LUTEIN DEFICIENT 5, chloroplastic, translation initiation factor IF-2 family protein, U1 small nuclear ribonucleoprotein 70 kDa and probable receptor-like serine/threonine-protein kinase At5g57670, among others. Significantly downregulated genes include those encoding Homeobox KN domain-containing protein, VIN3-like protein 2 isoform X2, CBL-interacting serine/threonine-protein kinase (CIPK), serine/threonine-protein kinase dst4-like isoform X1, and E3 ubiquitin-protein ligase BRE1-like, among others. In contrast, fewer DEGs were detected in comparisons among pc2, pc3, pc9, and pb29, with downregulated genes outnumbering upregulated genes in these comparisons. Conversely, more DEGs were identified between pc2, pc3, pc9, and CC84; the number of upregulated genes exceeded that of downregulated genes. A total of 3,636 DEGs were recorded across all 11 comparison groups.

The DEGs identified from the 11 differential expression analysis combinations were subjected to K-means hierarchical clustering analysis. As shown in Fig. 2b, all 3,636 DEGs were clustered into four expression patterns. Subcluster_1 contained 217 DEGs, in which all five transgenic poplar lines exhibited upregulated expression patterns relative to the wild type; expression levels in pc2, pc3, pc9, and pb29 were higher than those in CC84. Subcluster_2 comprised 391 DEGs, where all five transgenic lines showed downregulated expression patterns relative to the wild type. Subcluster_3 included 2,849 DEGs, for which only minor differences were detected between the transgenic lines and the wild type. Subcluster_4 consisted of 179 DEGs, where the five transgenic lines exhibited downregulated expression patterns compared to the wild type; pb29 showed the greatest downregulation.

#### Advanced UpSet plot analysis of DEGs

An UpSet plot comparing the five transgenic poplar 741 lines with the wild type across five groups is shown in Fig. 2c. Nineteen common DEGs were identified among all five comparison groups. One hundred twenty-seven common DEGs were shared by four comparison groups: pc2 vs. 741, pc3 vs. 741, pc9 vs. 741, and pb29 vs. 741. Only one common DEG was identified across the four comparison groups: pc2 vs. 741, pc3 vs. 741, pc9 vs. 741, and CC84 vs. 741. One hundred ten DEGs were exclusively shared by the pc2 vs. 741, pc3 vs. 741, and pc9 vs. 741 comparison groups but were not present in the other groups.

Compared to the wild type, the *Cry1Ac* gene was introduced into pc2, pc3, pc9, and pb29. Among the pc2 vs. 741, pc3 vs. 741, pc9 vs. 741, and pb29 vs. 741 comparison groups, 127 common DEGs were identified. The expression patterns of all these genes were consistent across the four comparison groups (Table S7). Most enzyme-encoding genes—such as *CHS*, *FLS*, *GDSL* esterase/lipase, auxin-binding protein, expansin, pathogenesis-related protein, and autophagy-related protein—as well as other functional genes, were upregulated. In contrast, genes encoding CBL-interacting serine/threonine-protein kinase 11, calmodulin-binding family protein, and putative disease resistance protein RGA3 were downregulated. The 127 DEGs identified may be involved in the response to *Cry1Ac* gene expression, thereby affecting plant growth.

Compared to the wild type, the *Cry3A* gene was introduced into pc2, pc3, pc9, and CC84. Among the pc2 vs. 741, pc3 vs. 741, pc9 vs. 741, and CC84 vs. 741 comparison groups, one common DEG potentially involved in the response to *Cry3A* gene expression was identified. This DEG was predicted to encode the metal–nicotianamine transporter gene *YSL3*. In all four comparison groups, this gene was upregulated.

The 110 DEGs shared among the pc2 vs. 741, pc3 vs. 741, and pc9 vs. 741 comparison groups, but they were not found in the other groups. These genes exhibited largely consistent expression patterns across the three comparison groups (Table S8). Among them, most enzymes—such as peroxidase, UDP-glucose 6-dehydrogenase, choline dehydrogenase, *GDSL* esterase/lipase, *ABCG14*, pathogenesis-related protein *PR-4*, fasciclin-like arabinogalactan protein 15, and fasciclin-like arabinogalactan protein 11—were upregulated. In contrast, the expression levels of *Fasciclin-like arabinogalactan protein 12*, cytokinin dehydrogenase, *bHLH143*, and *WRKY33* were downregulated. The 110 DEGS were likely involved in the response to secondary transformation, dual *Bt* gene expression, and their interaction.

The UpSet plot comparing the transgenic double *Bt* gene poplar 741 (pc) with pb29 across the pc2 vs pb29, pc3 vs pb29, and pc9 vs pb29 comparison groups is shown in Fig. 2d. In total, 16 common DEGs were identified among the three comparison groups (Table S9). Compared to pb29, the *Cry3A* gene was introduced into pc2, pc3, and pc9. Among these, the expression levels of genes encoding the 5’-3’ exoribonuclease family protein, CBL-interacting serine/threonine-protein kinase 21, and homeobox protein LUMINIDEPENDENS were upregulated. In contrast, the expression levels of genes encoding the SPINDLY family protein, alpha-farnesene synthase, and bHLH35 were downregulated. The 16 common DEGs shared by the three comparison groups may be involved in the response to secondary transformation, *Cry3A* gene expression, and gene interactions.

The UpSet plot of the three comparison groups between the double *Bt*-transgenic poplar 741 and CC84 is shown in Fig. 2e. In total, 267 common DEGs were identified among the three comparison groups (Table S10). Compared to CC84, the *Cry1Ac* gene was introduced into the pc2, pc3, and pc9 lines. The expression patterns of all these genes were consistent across the three comparison groups. Among them, upregulation of the genes encoding choline dehydrogenase, GDSL esterase/lipase, LRR receptor-like serine/threonine protein kinase, expansin, ABCG14, pathogenesis-related protein, SAUR family protein, laccase family protein, SPINDLY family protein, auxin-binding protein, bHLH48, and MYC2 was observed. The expression levels of genes encoding CBL-interacting protein kinase 11, E3 ubiquitin-protein ligase, cytokinin dehydrogenase, protein disulfide-isomerase A1, ABCG11/15, disease resistance protein, calmodulin-binding family protein, WRKY33, and bHLH35 were downregulated. The 267 common DEGs shared by the pc2 vs. CC84, pc3 vs. CC84, and pc9 vs. CC84 comparison groups may be involved in the response to secondary transformation, *Cry1Ac* gene expression, and gene interactions.

### Gene ontology functional classification and metabolic pathway enrichment analysis of DEGs

The GO functional classification results showed that among all comparison groups, the category with the most enriched DEGs was biological process, followed by molecular function; cellular component was the least enriched. In the comparisons of pc2, pc3, pc9, and pb29 with the wild type, we found that metabolic process, catalytic activity, and cell membrane were dominant in the biological process, molecular function, and cellular component categories, respectively. In the comparison of CC84 with 741, metabolic process, binding function, and cell membrane (including cell membrane part) were identified as dominant terms in the three major categories, respectively. Upon comparison of pc2, pc3, and pc9 with pb29 and CC84, metabolic process, binding function, and cell membrane remained predominant in the three major categories.

**Fig. 3:**
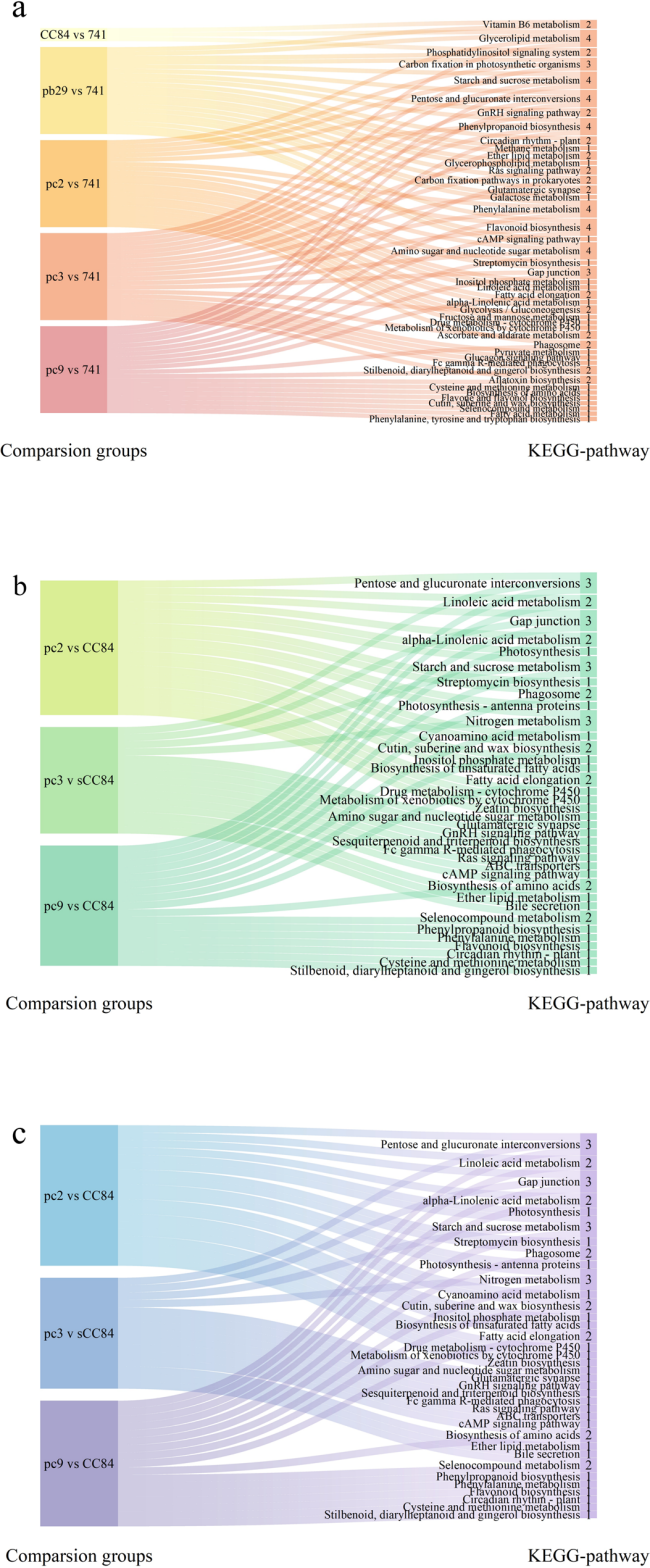
Significantly enriched KEGG metabolic pathways for each comparison group (top 20) Note: **a**: Top 20 significantly enriched KEGG metabolic pathways in the five comparison groups: PB29 vs 741, CC84 vs 741, PC2 vs 741, PC3 vs 741, and PC9 vs 741. **b**: Top 20 significantly enriched KEGG metabolic pathways in the three comparison groups: PC2 vs PB29, PC3 vs PB29, and PC9 vs PB29. **c**: Top 20 significantly enriched KEGG metabolic pathways in the three comparison groups: PC2 vs CC84, PC3 vs CC84, and PC9 vs CC84.

The significantly enriched KEGG metabolic pathways (top 20) for each comparison group are shown in Fig. 3. In the pc2 vs. 741, pc3 vs. 741, and pc9 vs. 741 comparison groups, 259, 213, and 309 DEGs, respectively, were annotated by KEGG; they participated in 147, 154, and 150 KEGG pathways. Most DEGs were associated with metabolism, followed by environmental information processing, organismal systems, genetic information processing, and cellular processes. Commonly enriched pathways among the three comparison groups included pentose and glucuronate interconversions, starch and sucrose metabolism, flavonoid biosynthesis, gap junction, phenylalanine metabolism, phenylpropanoid biosynthesis, and amino sugar and nucleotide sugar metabolism. In the comparison of pb29 with 741, 177 DEGs were annotated by KEGG and involved in 156 KEGG pathways. Most of these DEGs were associated with metabolism, followed by genetic information processing and organismal systems. Significantly enriched pathways included glycerolipid metabolism, carbon fixation in photosynthetic organisms, starch and sucrose metabolism, pentose and glucuronate interconversions, GnRH signaling pathway, and phenylpropanoid biosynthesis. In the comparison between CC84 and 741, 23 DEGs were annotated by KEGG and involved in 28 KEGG pathways. Most of these DEGs were related to metabolism, followed by genetic information processing and environmental information processing. Significantly enriched pathways included vitamin B6 metabolism, glycerolipid metabolism, and phosphatidylinositol signaling system. These results indicate significant differences in physiological metabolism between each of the pc2, pc3, pc9, pb29, CC84 and the wild type.

In the pc2 vs. pb29, pc3 vs. pb29, and pc9 vs. pb29 comparison groups, KEGG annotated 35, 21, and 151 DEGs, respectively; they participated in 47, 34, and 135 KEGG pathways. Significantly enriched pathways in the pc2 vs. pb29 and pc9 vs. pb29 comparisons included alpha-linolenic acid metabolism, antigen processing and presentation, and protein processing in the endoplasmic reticulum. Significantly enriched pathways in pc3 vs. pb29 included phenylalanine, tyrosine, and tryptophan biosynthesis, pentose phosphate pathway, and terpenoid backbone biosynthesis. Most DEGs were related to metabolism, followed by genetic information processing and environmental information processing. There were specific differences in physiological and metabolic levels between each of the three lines and pb29.

In the pc2 vs. CC84, pc3 vs. CC84, and pc9 vs. CC84 comparison groups, 245, 169, and 296 DEGs were annotated by KEGG; they were involved in 137, 147, and 155 KEGG pathways, respectively. Among the three comparison groups, common significantly enriched pathways included pentose and glucuronate interconversions, gap junction, starch and sucrose metabolism, and nitrogen metabolism. Most DEGs were related to metabolism, followed by genetic information processing. Significant differences in physiological metabolism were observed between each of the three lines and CC84.

### Transcription factor analysis

By searching the PlantTFDB, 230 transcription factors were identified among all DEGs and classified into 39 transcription factor families. The main families included *bHLH* (29), *AP2/ERF-ERF* (20), *MYB* (21), *WRKY* (19), *NAC*(18), *bZIP* (12), *Tify* (12), and *MYB-related* (10). The expression levels of all transcription factors are shown in Fig. S5. Compared to the wild type, pb29, and CC84, both upregulated and downregulated expression levels of transcription factors were observed in the transgenic double *Bt* gene poplar 741. The numbers of differentially expressed transcription factors in pc2, pc3, and pc9 compared to the wild type were 49, 38, and 72, respectively. These transcription factors were classified into 20, 18, and 25 families, respectively. Among them, the *WRKY*, *bHLH*, *MYB*, *AP2/ERF-ERF*, and *GARP-G2-like* families were most abundant. Additionally, the *WRKY* family was predominantly downregulated, whereas the *bHLH* family was mainly upregulated. In the comparison between pb29 and 741, 37 differentially expressed transcription factors were identified, belonging to 19 families. Among these, *bHLH*, *bZIP*, *MYB-related*, and *GARP-G2-like* were more abundant. In the comparison between CC84 and 741, seven differentially expressed transcription factors were detected, classified into four families. The numbers of differentially expressed transcription factors in pc2, pc3, and pc9 compared to pb29 were 17, 6, and 53, respectively. These factors were classified into 10, 5, and 20 families, respectively; the *bHLH* family was predominant. Additionally, most of these factors were downregulated. The numbers of differentially expressed transcription factors in pc2, pc3, and pc9 compared to CC84 were 81, 50, and 88, respectively. The *WRKY*, *bHLH*, *MYB*, *AP2/ERF-ERF*, and *NAC* families were more abundant. Among them, the *WRKY* family was mainly downregulated, whereas the *bHLH* family was predominantly upregulated.

### Validation of the RT-qPCR

The RT-qPCR results were compared to those obtained from transcriptome sequencing, as shown in Fig. S6. Although discrepancies were observed in measurements of the 10 DEGs between RT-qPCR and transcriptome sequencing, gene expression patterns were essentially consistent between the two methods, confirming the reliability of the transcriptome data.

### Plant hormone signaling pathways

**Fig. 4:**
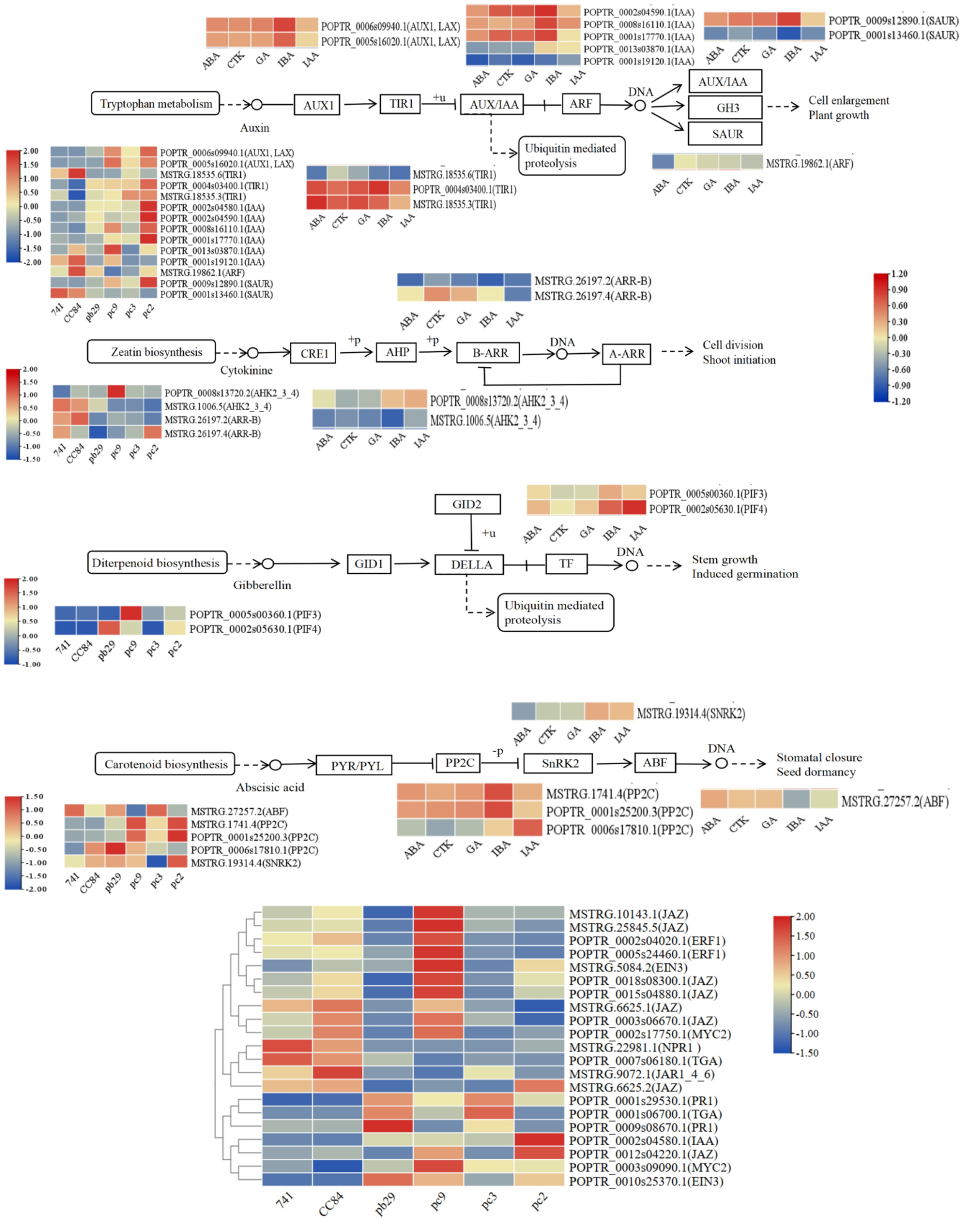
Heatmap of gene expression levels and regulation of hormone signaling pathways

Plant hormone signal transduction plays an important role in regulating plant growth and development. Compared to the wild type and transgenic single *Bt* gene poplar 741, gene expression in the plant hormone signal transduction pathway was partially altered in the transgenic double *Bt* gene poplar 741; The gene expression magnitude and pathway regulation direction are presented in Fig. 4.

In the auxin signal transduction pathway, two *AUX1/LAX* genes encoding auxin influx carriers (AUX1/LAX family) and one gene encoding a SAUR family protein were upregulated in pc2 vs. 741 and pc9 vs. 741, respectively. Three genes encoding auxin-responsive proteins of the AUX/IAA family were upregulated, and one SAUR gene was downregulated in pc2 vs. 741. In pb29 vs. 741, one transcriptional repressor response factor *TIR1* was downregulated. In pc9 vs. pb29, one *AUX1/LAX* gene was upregulated. In pc2 vs. CC84, pc3 vs. CC84, and pc9 vs. CC84, one auxin response factor ARF and one *SAUR* gene were upregulated. In pc2 vs. CC84 and pc9 vs. CC84, two *AUX1/LAX* genes, one transcription inhibitory response factor TIR1, and one *AUX/IAA* gene were upregulated (four genes in pc2 vs. CC84 and one gene in pc9 vs. CC84, respectively). In pc3 vs. CC84, two transcriptional repressor response factors TIR1 were upregulated, whereas two *AUX/IAA* genes were downregulated. In pc9 vs. CC84, one *ARF* gene and one *TIR1* gene were downregulated. Among them, *AUX1/LAX* genes (e.g., POPTR_0006s09940.1, POPTR_0005s16020.1) were positively correlated with IAA hormone content, while *ARF* gene (e.g., MSTRG.19862.1) was negatively correlated with IAA hormone content.

In the cytokinin signal transduction pathway, *ARR-B*, a gene encoding a two-component response regulator family protein, was downregulated in pc2 vs. 741, pb29 vs. 741, pc2 vs. pb29, and pc2 vs. CC84. In pc9 vs. 741, two genes encoding histidine kinase AHK2/3/4 exhibited differential expression patterns. Among them, *ARR-B* genes (e.g., MSTRG.26197.2, MSTRG.26197.4) were negatively correlated with CTK hormone content.

In the GA signal transduction pathway, upregulation of the phytochrome-interacting factor *PIF4* was observed in pc2 vs. 741, pc9 vs. 741, pc2 vs. CC84, and pc9 vs. CC84. Additionally, upregulation of the phytochrome-interacting factor *PIF3* was detected in pc9 vs. 741, pc9 vs. pb29, and pc9 vs. CC84. Among them, the *PIF3* gene (e.g., POPTR_0005s00360.1) was negatively correlated with GA hormone content.

In the ABA signaling pathway, the gene encoding protein phosphatase 2 C (*PP2C*) was upregulated in pc2 vs. 741, pc9 vs. 741, pb29 vs. 741, pc2 vs. CC84, and pc9 vs. CC84. The gene encoding serine/threonine-protein kinase SRK2 (*SnRK2*) was downregulated in pc3 vs. 741, pc3 vs. pb29, and pc3 vs. CC84. In pc9 vs. 741, the gene encoding the ABA response element binding factor (*ABF*) was downregulated. Among them, *PP2C* genes (e.g., MSTRG.1741.4, POPTR_0001s25200.3) were positively correlated with ABA hormone content; *SnRK2* gene (e.g., MSTRG.19314.4) was positively correlated with ABA hormone content; *ABF* gene (e.g., MSTRG.27257.2) was negatively correlated with ABA hormone content.

In the ethylene signaling pathway, the gene encoding ethylene-insensitive protein 3 (*EIN3*) was upregulated in pc2 vs. 741, pc9 vs. 741, pb29 vs. 741, pc2 vs. CC84, and pc9 vs. CC84. In pc9 vs. pb29, the ethylene-responsive transcription factor *ERF1* was upregulated.

In the jasmonic acid signaling pathway, the gene encoding the jasmonic acid ZIM domain protein (*JAZ*) was upregulated in pc2 vs. 741, pc2 vs. pb29, and pc9 vs. pb29. *JAZ* was downregulated in pc2 vs. 741 and pc2 vs. CC84. In CC84 vs. 741, *MYC2* expression was downregulated, whereas in pc2 vs. CC84, pc3 vs. CC84, and pc9 vs. CC84, *MYC2* expression was upregulated. The gene encoding jasmonic acid amino synthase (*JAR1*) was downregulated in pc9 vs. CC84.

In the salicylic acid signaling pathway, the gene encoding pathogenesis-related protein 1 (*PR1*) was upregulated in pc2 vs. 741, pc3 vs. 741, pc9 vs. 741, pb29 vs. 741, pc2 vs. CC84, pc3 vs. CC84, and pc9 vs. CC84. Additionally, differential expression of the transcription factor *TGA* and the regulatory protein gene *NPR1* was observed; downregulation was the predominant pattern.

### Phenylpropanoid biosynthesis pathway

The biosynthesis of phenylpropanoids is recognized as a crucial metabolic pathway in the synthesis of lignin monomers, which are considered to significantly influence plant growth. Unlike the wild type and transge nic single *Bt* gene poplar 741, DEGs annotated to the phenylpropanoid biosynthesis pathway were identified in transgenic double *Bt* gene poplar 741 (Table 1).

**Table 1:** Enriched DEGs in the phenylpropanoid biosynthesis pathway

Comparative groups	DEGs
pc2 vs. 741	*POD* (7, up), *PAL* (1, up), *bglB* (1, up), *CAD* (1, down), *4CL* (1, down), *HCT* (1, down)
pc3 vs. 741	*POD* (4, up), *PAL* (1, up), *bglB* (2, up), *CCoAOMT* (1, up), *CYP98A3* (1, up), *4CL* (1, up), *POD* (1, down), *HCT* (1, down)
pc9 vs. 741	*POD* (12, up), *PAL* (3, up), *bglB* (2, up), *CCoAOMT* (2, up), *4CL* (3, up), *CYP98A3* (1, up), *CSE* (2, up), *CCR* (1, down)
pb29 vs. 741	*POD* (4, up), *PAL* (1, up), *bglB* (2, up), *CAD* (1, up), *4CL* (1, up), *CYP98A3* (1, up)
pc2 vs. pb29	*4CL* (1, down)
pc9 vs. pb29	*POD* (2, up), *bglB* (1, up)
pc2 vs. CC84	*POD* (4, up), *bglB* (1, up), *4CL* (1, down), *CCR* (1, down)
pc3 vs. CC84	*POD* (2, up), *bglB* (1, up), *CYP98A3* (1, up), *CAD* (2, down), *CCR* (1, down)
pc9 vs. CC84	*POD* (10, up), *PAL* (3, up), *CCoAOMT* (2, up), *bglB* (1, up), *4CL* (4, up), *CSE* (2, up), *CYP98A3* (1, up), *CCR* (1, down)

In the pc2 vs. 741 comparison, 12 DEGs were annotated to the phenylpropanoid biosynthesis pathway, including upregulated expression of peroxidase (*POD*), phenylalanine ammonia-lyase (*PAL*), and beta-glucosidase (*bglB*) genes, and downregulated expression of cinnamyl alcohol dehydrogenase (*CAD*), 4-coumaroyl-CoA ligase (*4CL*), and shikimate hydroxycinnamoyltransferase (*HCT*) genes. In pc3 vs. 741, 12 DEGs were annotated in the phenylpropanoid biosynthesis pathway (Table 1). Genes including *POD*, *4CL*, *bglB*, caffeoyl-CoA O-methyltransferase (*CCoAOMT*), coumaroyl quinolone 3’-monooxygenase (*CYP98A3*), and *PAL* were upregulated, whereas *POD* and *HCT* genes were downregulated. In pc9 vs. 741, 26 DEGs were annotated to the phenylpropanoid biosynthesis pathway (Table 1). Genes such as *POD*, *4CL*, *bglB*, *CCoAOMT*, *CYP98A3*, *PAL*, and caffeoyl shikimate esterase (*CSE*) were upregulated, whereas cinnamoyl-CoA reductase (*CCR*) was downregulated. In pb29 vs. 741, 10 DEGs were annotated to the phenylpropanoid biosynthesis pathway; the *POD*, *4CL*, *bglB*, *CYP98A3*, *PAL*, and *CAD* genes were upregulated.

In pc2 vs. pb29, the expression of the *4CL* gene was downregulated. In pc9 vs. pb29, *bglB* was downregulated and *POD* was upregulated. In pc2 vs. CC84, *POD* and *bglB* were upregulated, whereas *4CL* and *CCR* were downregulated. In pc3 vs. CC84, *POD*, *bglB*, and *CYP98A3* were upregulated; *CAD* and *CCR* were downregulated. In pc9 vs. CC84, 22 DEGs were annotated in the phenylpropanoid biosynthesis pathway, with upregulated expression of *POD*, *CCoAOMT*, *bglB*, *4CL*, *PAL*, *CSE*, and *CYP98A3*, and downregulated expression of *CCR*. Some of these DEGs were inferred to partially promote lignin synthesis, thereby enhancing cell wall strength and rigidity, which would improve the plant’s protective and defensive capabilities against damage.

### WGCNA results

#### Co-expression network construction and correlation analysis between gene modules and trait matrices

The expression data of all genes in the transcriptome were used to generate a sample clustering diagram of gene expression levels (Fig. S7a). Based on the screening of weight values, β = 17 was ultimately selected to construct the network (Fig. S7b). The dynamic tree cut method was used to merge modules with similar expression patterns, resulting in a total of 33 co-expression modules (Fig. S7c). The number of genes in each module was statistically analyzed, with the results shown in Fig. S7d. The grey module, which contained 10,045 genes, was defined as a gene set not assigned to any specific module. The five largest modules, ranked by the number of assigned genes, were turquoise (8,300 genes), blue (6,612 genes), brown (3,706 genes), yellow (2,567 genes), and green (2,560 genes). Interactions between modules were visualized using the TOM matrix (Fig. S7e). The heatmap displayed a diagonal mirrored pattern, indicating that each module was mutually independent. This demonstrated a high degree of independence between modules and relative consistency in gene expression within each module.

**Fig. 5:**
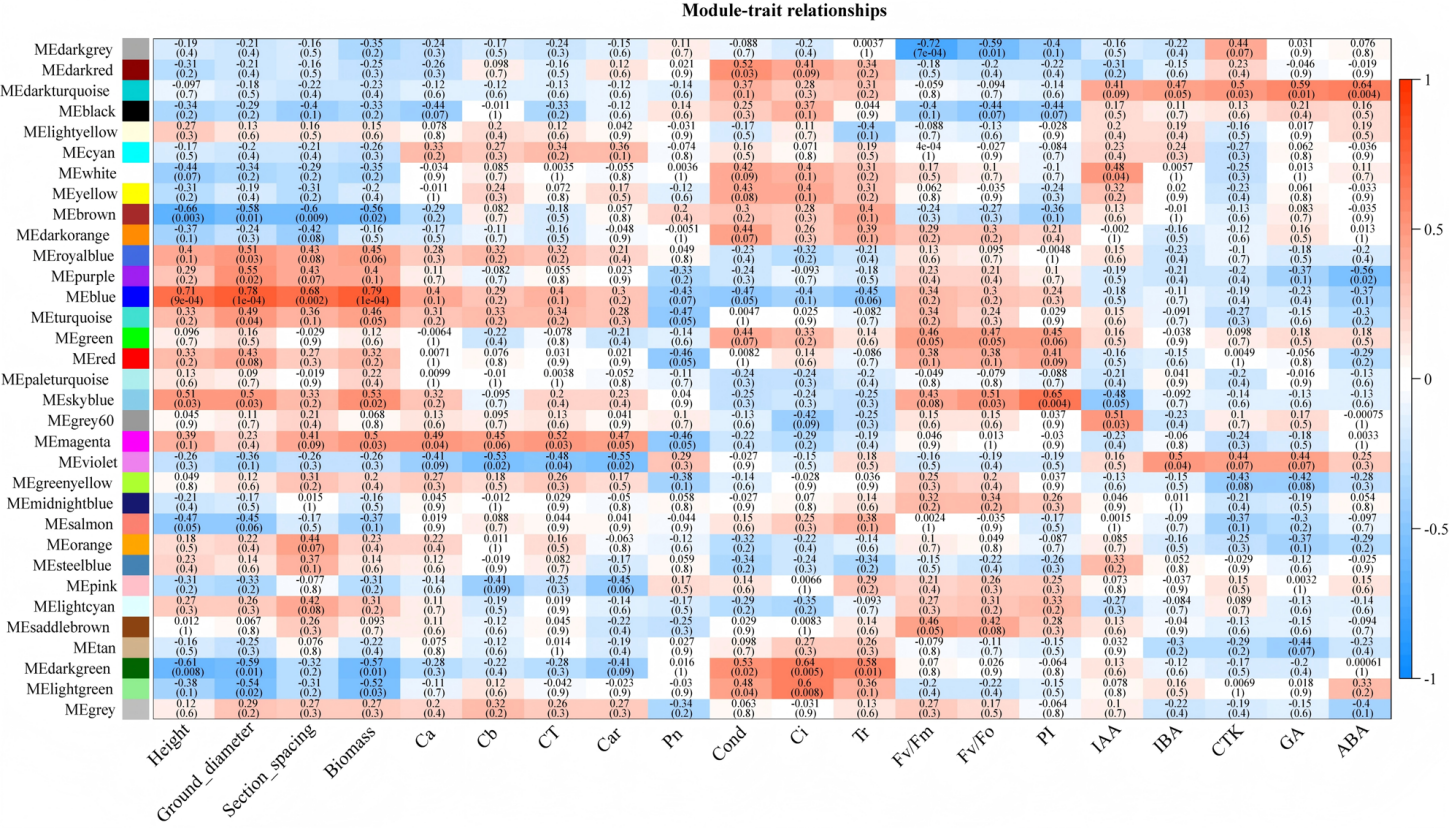
Association analysis of gene co-expression network modules with phenotype traits. Note: The horizontal axis represents physiological and biochemical traits at different processing times, whereas the vertical axis represents the feature vectors of each module. The red grid indicates a positive correlation between traits and modules; the blue grid indicates a negative correlation

The characteristic values of each sample across various modules were correlated with the phenotypic matrix to identify modules associated with specific traits. From the module–trait correlation heatmap (Fig. 5), some modules were strongly correlated with particular traits. For example, genes in the blue module were significantly positively correlated with plant height, ground diameter, internode length, and biomass, with correlation coefficients of 0.71, 0.78, 0.68, and 0.79, respectively (*p* < 0.01), all of which were highly significant. Genes in the brown module were significantly negatively correlated with plant height, ground diameter, internode length, and biomass, with correlation coefficients of − 0.66, − 0.58, − 0.60, and − 0.56, respectively (*p* < 0.01). The magenta module was significantly positively correlated with photosynthetic pigments (Ca, CT, and Car). The dark turquoise module was significantly positively correlated with plant hormones (IBA, CTK, GA, and ABA). The dark green module was significantly negatively correlated with plant height, ground diameter, and biomass; it was significantly positively correlated with *Cond*, *Ci*, and *Tr*.

#### Selection of target gene modules and core genes

**Fig. 6:**
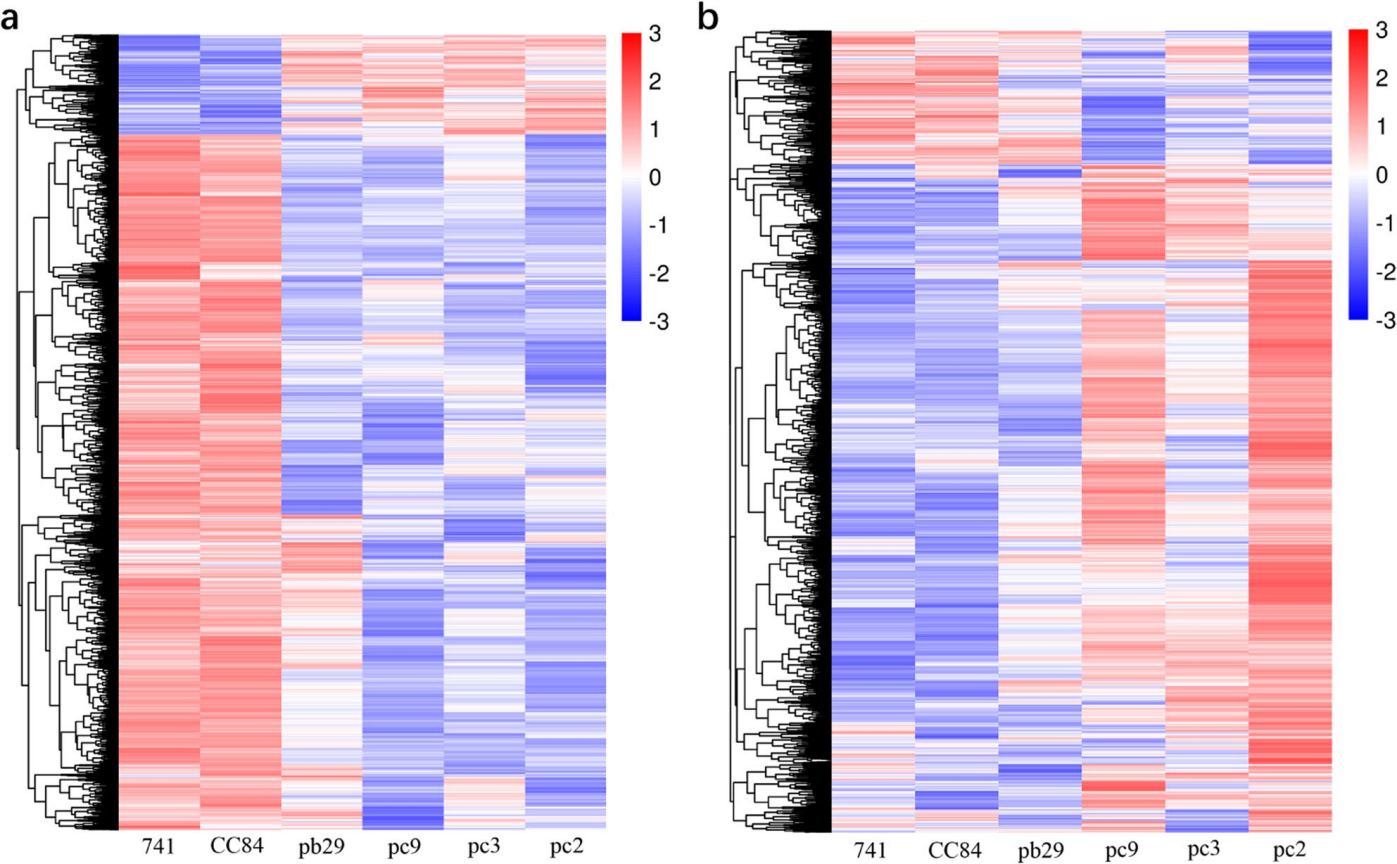
Heat map of gene expression levels in the blue (**a**) and brown (**b**) modules

Based on the association heatmap between gene modules and traits, the blue and brown modules, which were strongly correlated with plant height, ground diameter, internode length, and biomass, were selected as target modules. Expression heatmaps were plotted for all genes in each module to analyze their expression trends across different samples, with the results shown in Fig. 6. In the blue module, higher gene expression levels were observed in 741 and CC84; lower expression levels were recorded in pb29 and the transgenic double *Bt* lines (Fig. 6a). In the brown module, the transgenic double *Bt* lines showed higher gene expression levels, whereas the other lines exhibited lower levels (Fig. 6b).

**Fig. 7:**
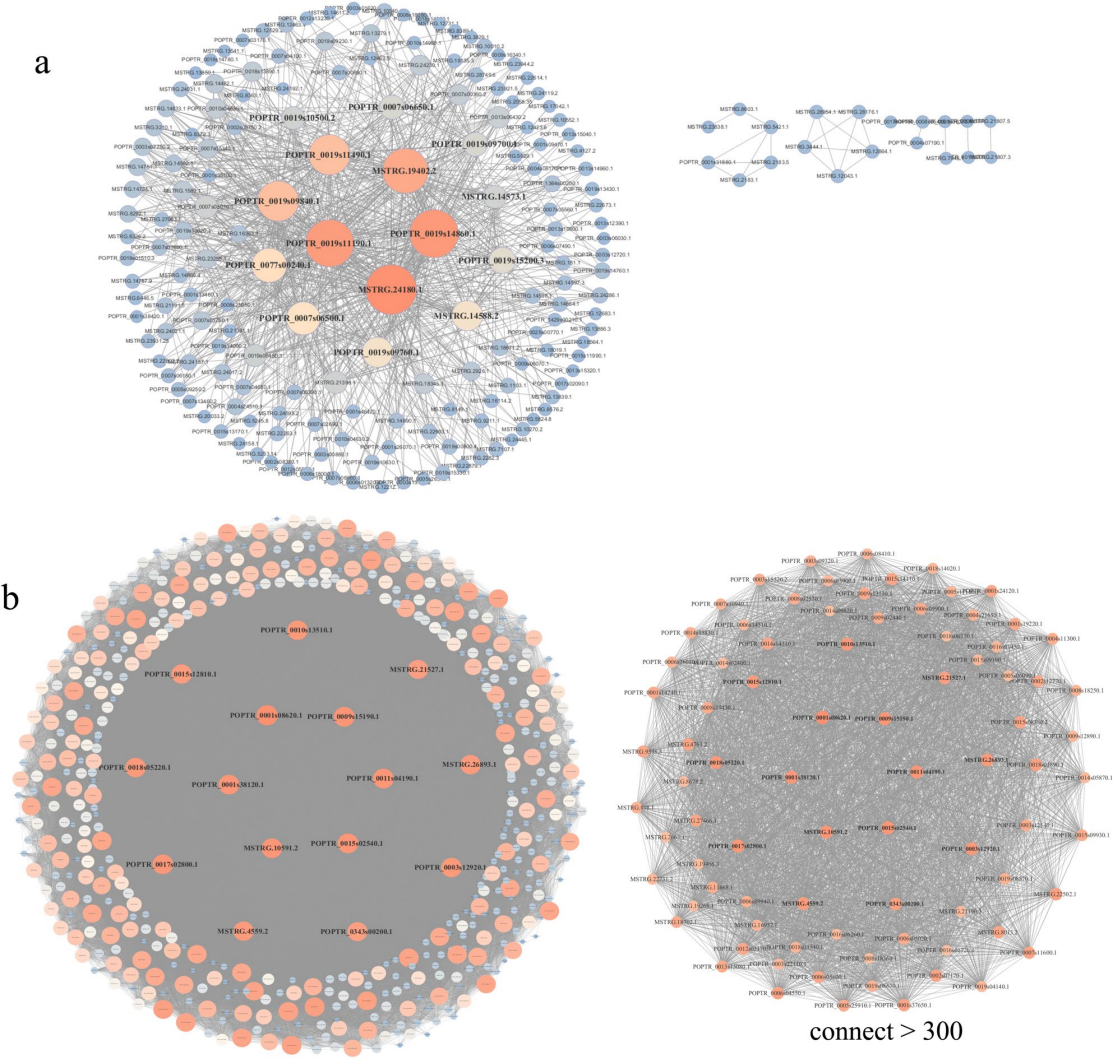
Co-expression networks of DEGs in the blue (**a**) and brown (**b**) modules

DEGs in the blue module were used to construct a co-expression network, and the results are shown in Fig. 7a. Genes with the strongest connectivity (top 15) were defined as hub genes (Table S11). These included genes encoding somatic embryogenesis receptor kinase 1, CBL-interacting serine/threonine-protein kinase 11, protein disulfide-isomerase A1, disease resistance-like protein DSC1, apoptosis-antagonizing transcription factor, and programmed cell death protein 2-like. Expression levels of the top 15 hub gnes in this module were significantly positively correlated with four growth traits (plant height, ground diameter, section spacing, and biomass), with correlation coefficients ranging from *r* = 0.51 to 0.78 (Fig. S8a). Key genes included CBL-interacting serine/threonine-protein kinase 11 and disease resistance-like protein DSC1, which showed the strongest positive correlations with ground diameter (*r* = 0.78) and biomass (*r* = 0.78). Splicing factor 3B subunit 4 also exhibited strong associations with plant height (*r* = 0.69) and biomass (*r* = 0.78). Protein disulfide-isomerase A1 showed the strongest positive correlation with section spacing (*r* = 0.66). CBL-interacting serine/threonine-protein kinase 11 and disease resistance-like protein DSC1 exhibited the strongest positive correlations with ground diameter (*r* = 0.78).

In the brown module, a co-expression network was also constructed from the DEGs and is shown in Fig. 7b. The top 15 highly connected genes were defined as hub genes (Table S11), including genes encoding choline dehydrogenase, glucomannan 4-beta-mannosyltransferase 2, vacuolar protein 8, protein ASPARTIC PROTEASE IN GUARD CELL 1, cytochrome P450 CYP86A4S, putative cyclin-dependent serine/threonine-protein kinase, 3-ketoacyl-CoA synthase, ABC transporter C family member 15, fatty alcohol: caffeoyl-CoA acyltransferase, BAHD acyltransferase DCR, and protein LONGIFOLIA 1-like isoform X1. Expression levels of the top 15 hub gnes in this module were significantly negatively correlated with four growth traits (plant height, ground diameter, section spacing, and biomass), with correlation coefficients ranging from *r* = − 0.45 to − 0.68 (Fig. S8b). Key features included the strongest negative correlations for vacuolar protein 8 with plant height (*r* = − 0.68) and internode length (*r* = − 0.60), and stably strong negative correlations for predicted protein LONGIFOLIA 1-like isoform X1 with plant height (*r* = − 0.63) and internode length (*r* = − 0.63). Glucomannan 4-beta-mannosyltransferase 2 and predicted heavy metal-associated isoprenylated plant protein 7 exhibited the strongest negative correlation with ground diameter (*r* = − 0.56). Glucomannan 4-beta-mannosyltransferase 2 showed the strongest negative correlation with biomass (*r* = − 0.57). The identification of core genes lays a foundation for further in-depth analysis of the regulatory mechanisms by which *Bt* genes influence the growth of poplar 741.

## Discussion

### Unexpected effects and potential causes of exogenous *Bt* gene insertion

The introduction of exogenous genes has partly improved the traits of target plants, but it may also lead to unintended effects [37, 38]. The products of exogenous gene expression are not essential for the growth and development of the parental plants, and their expression requires energy consumption. This disrupts the original balance between energy supply and demand in the plant, thereby affecting normal physiological metabolism, leading to phenotypic differences, and ultimately resulting in unintended effects [39, 40]. The overexpression of exogenous Cry1Ac protein in tobacco (*Nicotiana tabacum*) and cotton (*Gossypium hirsutum*) cytoplasm has been reported to affect normal plant growth; if the expression level is further increased, it may cause more pronounced phenotypic differences [41]. Previous studies have shown that tobacco plants transformed with dual *Cry1Ac* genes exhibit reduced height compared to both plants transformed with a single *Cry1Ac* gene and control plants. This is potentially because high expression levels of Cry1Ac toxin protein exert an inhibitory effect on plant growth [42]. Our research team utilized a plant binary expression vector to simultaneously transfer dual *Bt* genes into poplar trees, generating transgenic poplars resistant to both Lepidoptera and Coleoptera. However, the expression level of the Cry1Ac toxin protein was relatively low, and resistance to later-instar Lepidoptera larvae was poor [1, 2]. Nevertheless, the growth of transgenic poplar trees containing dual *Bt* genes was not significantly affected [43].

Studies have shown that the use of the constitutive strong promoter CaMV35S may lead to dwarfism in transgenic plants. Even when the plant does not require it, the target gene driven by this promoter can be strongly expressed, disrupting normal gene regulation and metabolic pathways, thus affecting the plant’s growth and development [44]. The present study showed that, in transgenic double *Bt* gene poplar 741, the *Cry1Ac* and *Cry3A* genes were both driven by the CaMV35S promoter. These exogenous genes were continuously transcribed and translated in the recipient plant, producing large amounts of Cry1Ac and Cry3A toxic proteins. As a result, the recipient plant may have altered its normal gene regulation and metabolic pathways to mitigate the impact of exogenous gene expression.

### Mechanistic link between dwarf phenotype and phytohormones in dual *Bt* transgenic poplar 741

The most important feature observed in the transgenic double *Bt* gene poplar 741 compared to the wild type in this study is dwarfism. The dwarf phenotype is primarily caused by a decrease in cell number, a reduction in intercellular space, and shorter cell length. Complex interactions between various plant hormones collectively influence plant growth and development [45]. This study used potted seedlings under controlled conditions; however, research on long-lived woody plants is limited by ecological and developmental relevance, as seedling-stage growth inhibition cannot reflect long-term field performance, and practical application of the results requires further validation in natural habitats. Hu Baozhong et al. [46] found that the ABA and IAA contents in various tissues of dwarf cucumber (*Cucumis sativus* var. *humilis*) were significantly higher than those of creeping cucumber (*Cucumis sativus* var. *Vulgaris*) during the early growth stage. In our study, the IAA, IBA, and ABA contents in the leaves of the transgenic double *Bt* gene poplar 741 lines were higher than those in the wild type, and the GA content of the two lines was also higher than that of the wild type. Generally, low concentrations of auxin promote plant growth. However, beyond an optimal concentration, auxin can stimulate ethylene production and disrupt the internal physiological balance, thereby inhibiting growth. Furthermore, the growth of the transgenic double *Bt* gene poplar 741 was slower than that of the wild type, possibly due to auxin concentrations exceeding the optimal threshold, which inhibited growth and resulted in reduced growth rates.

In addition to the types and levels of plant hormones, plant cell elongation and stem growth are intricately regulated by transcription factors involved in plant hormone signaling pathways [47–50]. Fenglan [51] reported differential expression of multiple genes related to hormone signal transduction in tall and short-stem castor (*Ricinus communis*) plants. In the present study, key genes involved in the biosynthesis, metabolism, and signal transduction of hormones such as auxin, cytokinin, brassinosteroids, and jasmonic acid—including *AUX/LAX*, *AUX/IAA*, *SAUR*, and *JAZ*—showed significantly altered expression levels within poplar 741 lines transformed using the double *Bt* gene. These changes may influence plant growth and could be a major cause of the observed dwarfism. Overall, the integration and efficient expression of *Bt* genes led to changes in the expression of genes related to plant hormone signaling pathways and altered endogenous hormone content in transgenic double *Bt* gene poplar 741. This hormonal imbalance may underlie the observed dwarfism and reduced growth potential.

### Differentially Expressed Gene (DEG) analysis and functional interpretation of key hub genes

In this study, the transgenic double *Bt* gene poplar 741 exhibited a greater number of DEGs compared to the wild type and CC84. The number of DEGs associated with pb29 was relatively small, which may be related to the secondary transformation of exogenous genes. The insertion and expression of exogenous genes can influence phenotypic traits, nutrient composition, and secondary metabolite levels in recipient plants [52], and these effects are often accompanied by changes in the transcriptional regulation of upstream genes. Compared to the wild type and CC84, genes encoding auxin-binding protein, pathogenesis-related protein, expansin, GDSL esterase/lipase, laccase, and SPINDLY family proteins were upregulated in the transgenic double *Bt* gene poplar 741, whereas genes encoding CBL-interacting protein kinase 11, disease resistance proteins, calmodulin-binding family proteins, protein disulfide-isomerase A1, and others were downregulated. Various transcription factor families also showed differential expression, and these gene expression changes may be part of the plant’s response to *Cry1Ac* gene expression, which is closely linked to the inhibition of plant growth. Among them, the genes encoding probable receptor-like serine/threonine-protein kinase At5g57670 [53] was significantly upregulated in transgenic lines. As a core negative regulator of growth inhibition, its overactivation leads to energy shift toward defense responses, and knockdown of its expression using RNAi or CRISPR-Cas9 technology can directly alleviate the dwarf phenotype. Both CBL-interacting serine threonine-protein kinase (CIPK) [54] and Homeobox KN domain [55] genes were significantly downregulated, regulating growth signal transduction and meristem function, respectively; overexpression of these two genes can restore growth signal transmission and meristem activity, thereby alleviating growth retardation and dwarfism. The genes encoding E3 ubiquitin-protein ligase BRE1-like [56] served as an alternative target, and moderate upregulation of its expression can restore the homeostasis of protein metabolism and chromatin modification, assisting in optimizing the molecular environment for growth. In summary, the growth inhibition side effects of dual *Bt* transgenic Poplar 741 can be maximally alleviated through the bidirectional regulation of inhibiting core negative regulatory kinases and enhancing key positive growth regulatory genes, combined with the auxiliary role of alternative targets. The study also found that there were fewer DEGs involved in the response to *Cry3A* gene expression across different comparison groups. Compared to the wild type, the growth of pb29 was less severely affected, suggesting that the growth inhibition observed in transgenic double *Bt* gene poplar 741 is due to the high expression of the *Cry1Ac* gene and its interaction with the *Cry3A* gene.

Based on the WGCNA results, hub genes were selected from the key target gene modules (blue and brown). CBL-interacting serine/threonine-protein kinase, also known as calcineurin B-like (CBL) interacting protein kinase (CIPK), is a unique type of Ser/Thr protein kinase found in plants. The CBL/CIPK complex is involved in plant responses to osmotic stress, ABA signaling, low temperature, and overall growth and development. ABCG is the largest subfamily of ABC transporters; these play major roles in exporting secondary metabolites and hormones induced by key biological stresses such as jasmonic acid and salicylic acid. They also respond to low temperature and ABA stress [57]. In this study, the expression level of ABCG15 was increased in transgenic double *Bt* gene poplar 741, suggesting a role in the response to hormones such as ABA. The identification of hub genes lays a foundation for further in-depth analysis of the molecular mechanisms through which *Bt* genes regulate the growth of poplar 741. Notably, only three biological replicates were included in the RNA-seq and WGCNA analyses in this study, which was a reasonable sample size with high comparability achievable under current conditions, constrained by the strict screening of homogeneous materials, high cultivation cost, and long growth cycle required for dual *Bt* transgenic poplar 741. Although analytical rigor was ensured using the DESeq2 algorithm, stringent filtering thresholds, and optimized WGCNA network parameters, this study still has limitations such as insufficient statistical power and relatively lower stability of co-expression network topology compared with large-sample studies. Therefore, the differentially expressed genes and co-expression modules identified herein only represent the core gene set with the most significant changes associated with the target traits, and are intended to provide candidate genes and scientific hypotheses for further research.

### Integrated mechanisms of growth inhibition in dual *Bt* transgenic poplar 741 and future research directions

A comprehensive analysis of phenotypic variation and transcriptomic changes in poplar 741 transformed with the double *Bt* gene revealed that integration, efficient expression, and gene interactions altered the expression patterns of multiple growth-related genes across various metabolic pathways—including plant hormone signal transduction, porphyrin and chlorophyll metabolism, and flavonoid biosynthesis. The accumulation of *Bt* toxin proteins, especially *Cry1Ac*, appears to disrupt multiple metabolic pathways, resulting in decreased chlorophyll content, increased auxin and ABA levels, and hormonal imbalances that impair normal plant growth. These may be the primary factors contributing to the significant growth inhibition observed in transgenic double *Bt* gene poplar 741. The copy number of the exogenous *Bt* gene directly affects its transcription and expression level, and higher expression may lead to increased cellular metabolic consumption; meanwhile, the insertion site of the transgene could cause position effects and alter the expression of adjacent endogenous genes. Therefore, further exploration of *Bt* protein function and the mechanisms leading to unintended effects will be the focus of future research. A study of genetically modified rice revealed that the expression of exogenous *Cry1Ab/c* genes can lead to interactions with endogenous proteins. Using yeast two-hybrid, bimolecular fluorescence complementation, and immunoprecipitation techniques, researchers confirmed that the Cry1Ab/c protein interacts with endogenous proteins involved in photosynthesis and stress resistance, leading to phenotypic differences [33]. The flowering time of the insect-resistant rice Huahui-1, transformed with the *Cry1Ab/c* gene, was later than that of its parent line, Minghui-63, when cultivated in both farmland and saline-alkali soil. These same techniques demonstrated that exogenous Cry1Ab/c delays flowering in insect-resistant transgenic rice by interacting with Hd3a anthocyanin [58]. The integration and expression of exogenous genes can thus have specific unintended effects on recipient plants. In future research, various technical approaches can be used to explore the functions of endogenous proteins that interact with exogenous proteins and to identify these interactions based on phenotypic traits. This will help clarify the molecular mechanisms underlying phenotypic variation in transgenic plants and provide a theoretical basis for the safety assessment and rational utilization of transgenic crops.

## Conclusions

In this study, differences in phenotypic traits and transcriptional profiles were analyzed among secondary-transformed double *Bt* gene poplar 741, transgenic single *Bt* gene poplar 741, and the wild type. Compared to both the wild type and the transgenic single *Bt* gene poplar 741, the plant height, ground diameter, and biomass of the transgenic double *Bt* gene poplar 741 were significantly reduced. The phenotype showed distinct changes, including reduced plant height, slow growth, and weakened growth potential. Photosynthetic parameters (*Ci*, *Cond*, and *Tr*) were significantly increased, whereas photosynthetic pigment content in some lines and chlorophyll fluorescence parameters decreased. Additionally, the levels of IAA, IBA, and ABA were elevated. These findings indicated significant alterations in both photosynthesis and endogenous hormone levels in the transgenic double *Bt* gene poplar 741. Furthermore, the number of DEGs between transgenic double *Bt* gene poplar 741 and the wild type or CC84 was higher than that observed for pb29. The common DEGs in pc2 vs. 741, pc3 vs. 741, pc9 vs. 741, and pb29 vs. 741 may be involved in the response to *Cry1Ac* gene expression. Common DEGs found in pc2 vs. 741, pc3 vs. 741, and pc9 vs. 741, but not in other comparison groups, may be involved in the response to secondary transformation, double *Bt* gene expression, and their interaction. These DEGs are mainly associated with metabolic processes such as pentose and glucuronate interconversions, starch and sucrose metabolism, flavonoid biosynthesis, and plant hormone signal transduction. In the plant hormone signal transduction and phenylpropanoid biosynthesis pathways, differential expression of several key regulatory genes—such as *AUX1/LAX*, *AUX/IAA*, *SAUR*, *ARF*, *PIF*, *PR1*, *4CL*, *bglB*, *CCoAOMT*, *CYP98A3*, and *PAL*—may influence plant growth by affecting hormone signal transduction and lignin synthesis. Based on WGCNA, the blue and brown gene modules (significantly correlated with plant height, ground diameter, internode spacing, and biomass) were identified. Candidate hub genes encoding CBL-interacting serine/threonine-protein kinase 11, protein disulfide-isomerase A1, disease resistance-like protein DSC1, CYP86A4S, ABCG15, and other growth-related genes were also identified. Based on physiological and transcriptomic characteristics, we speculate that the high expression and gene interaction of the double *Bt* genes affected the differential expression of numerous growth-related genes in pathways such as plant hormone signal transduction and phenylpropanoid biosynthesis. This may have resulted in metabolic disorders, which are likely the main cause of the phenotypic variation observed in the secondary-transformed double *Bt* gene poplar 741. This study provides a reference for further investigation into the unintended effects of transgenic plants.

## Supplementary Information

Supplementary Material 1.

Supplementary Material 2.

## Data Availability

The raw data of transcriptome sequencing generated during the current study have been deposited in the National Center for Biotechnology Information (NCBI) database. The relevant data can be accessed via the following accession number PRJNA1301531.
