# Supplementary material for: Transcriptome sequencing and weighted correlation network analysis reveal the molecular mechanism of growth variation in highly resistant transgenic double Bt poplar
Source: BMC Plant Biol. 2026 Feb 26;26:598. doi: 10.1186/s12870-026-08431-y (PMC13041089; doi:10.1186/s12870-026-08431-y)
Supplement: Supplementary file 2 — Supplementary Material 2. [file 12870_2026_8431_MOESM2_ESM.docx]

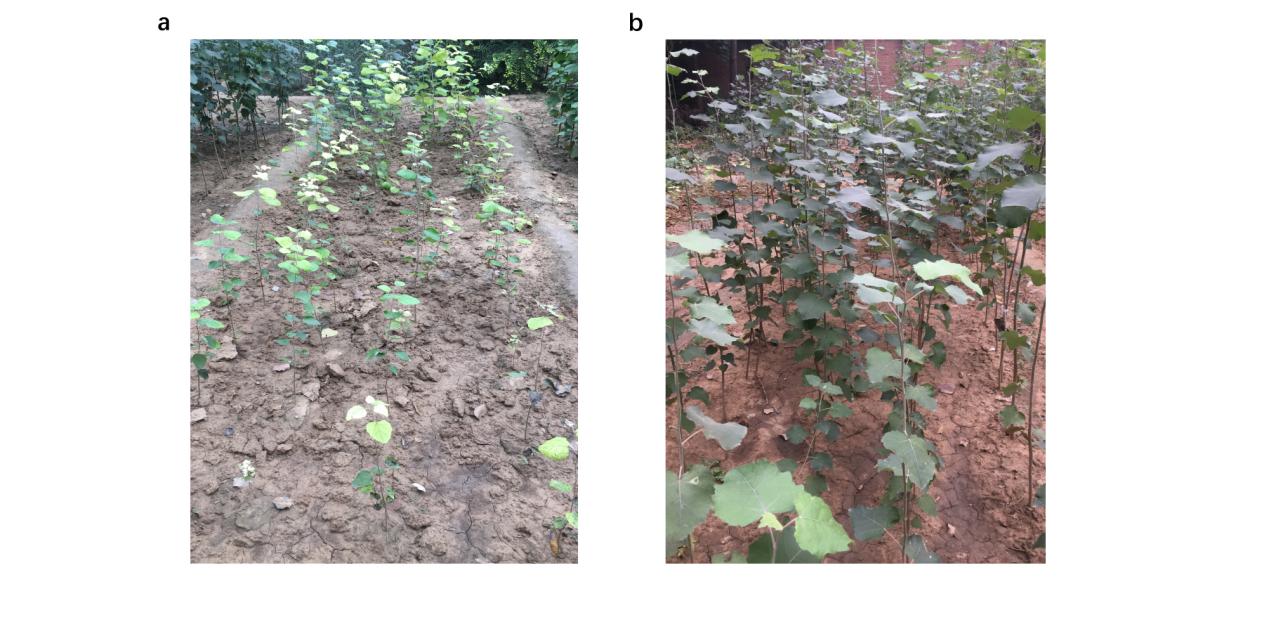


Supplementary Figure S1 The growth performance of the secondary-transformed double *Bt* gene poplar 741 (planted in the experimental field by our research group)


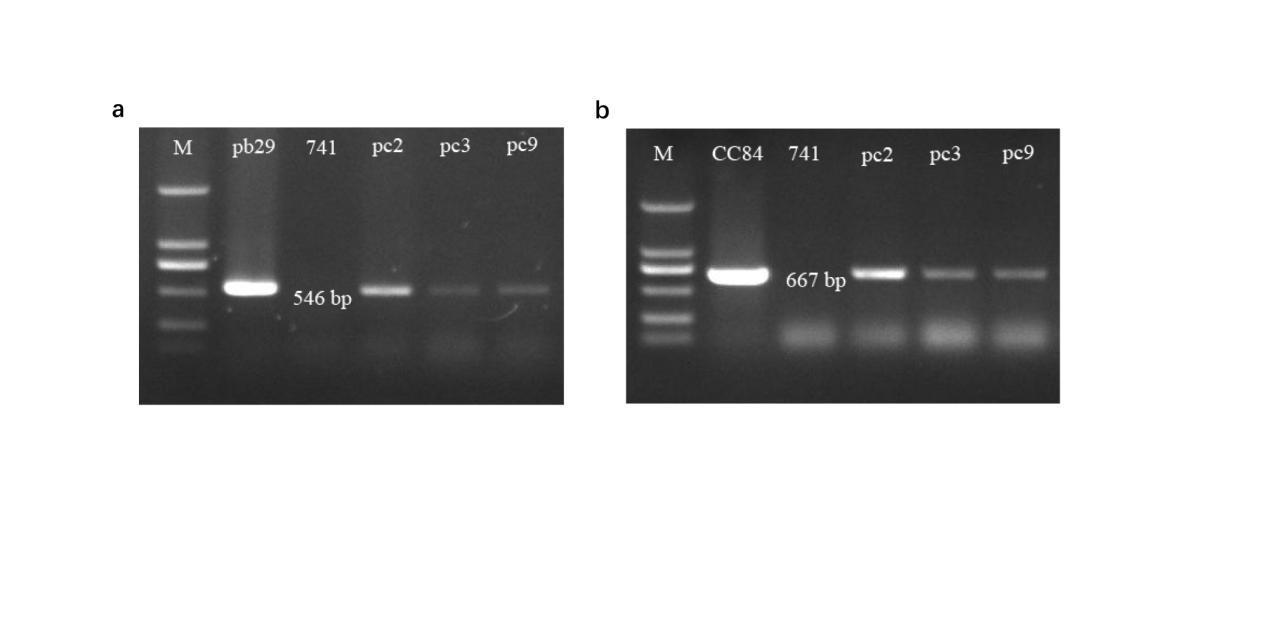


Supplementary Figure S2 PCR detection of the double *Bt* gene


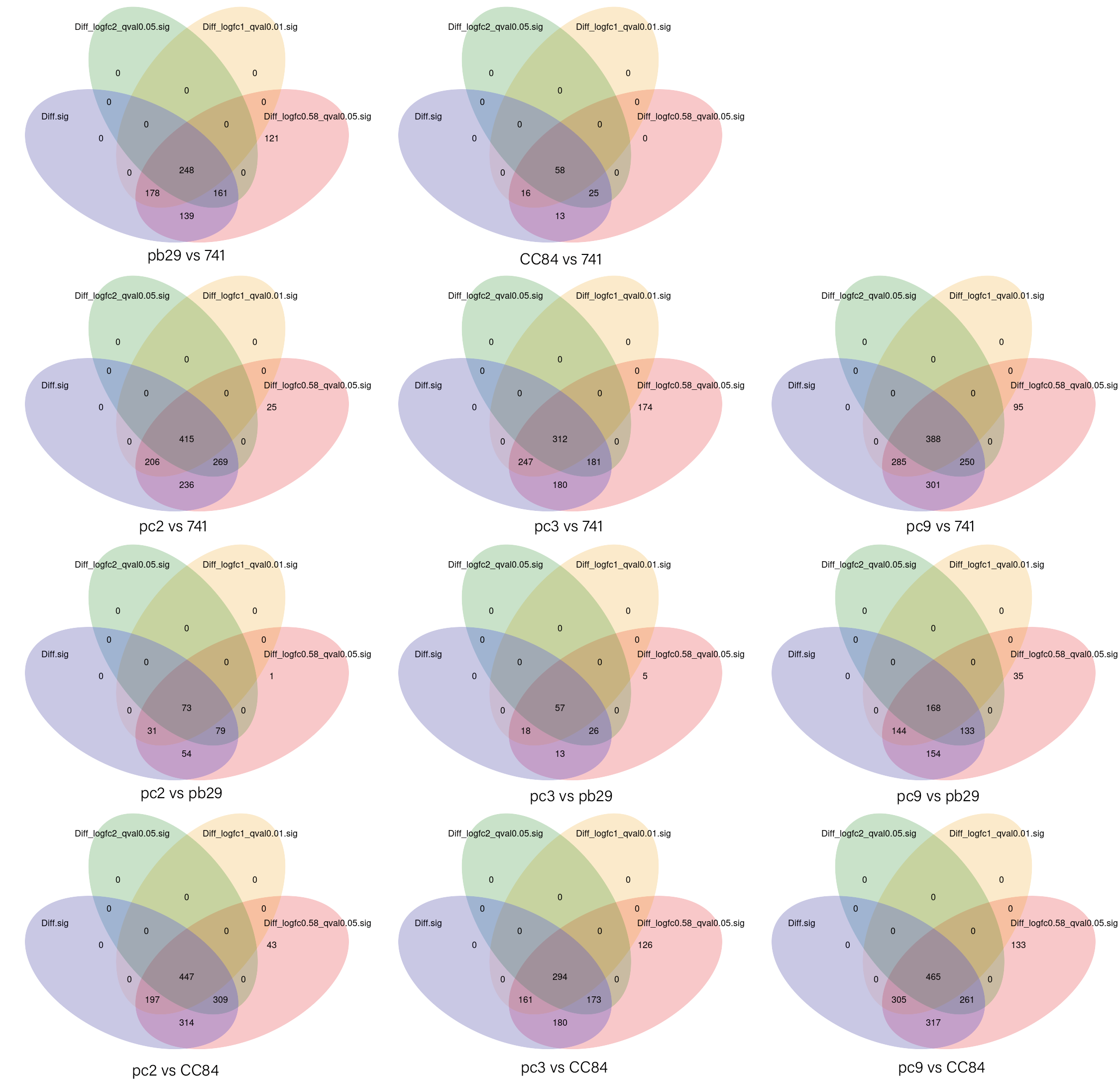


Supplementary Figure S3 Multi-threshold gradient sensitivity analysis


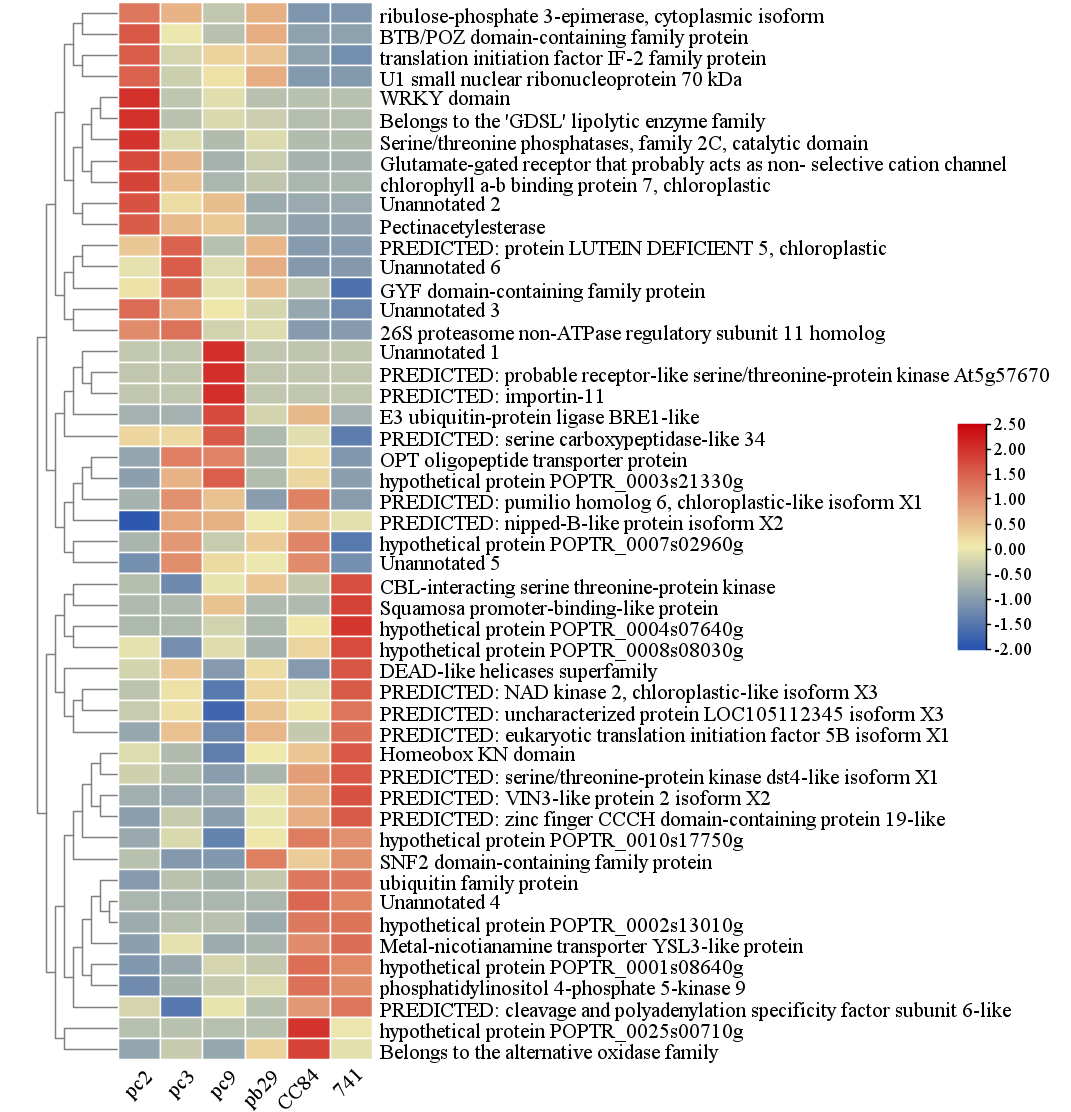


Supplementary Figure S4 The heatmap of the top 50 most significantly up- and down-regulated DEGs between pc2, pc3, pc9 and wild-type


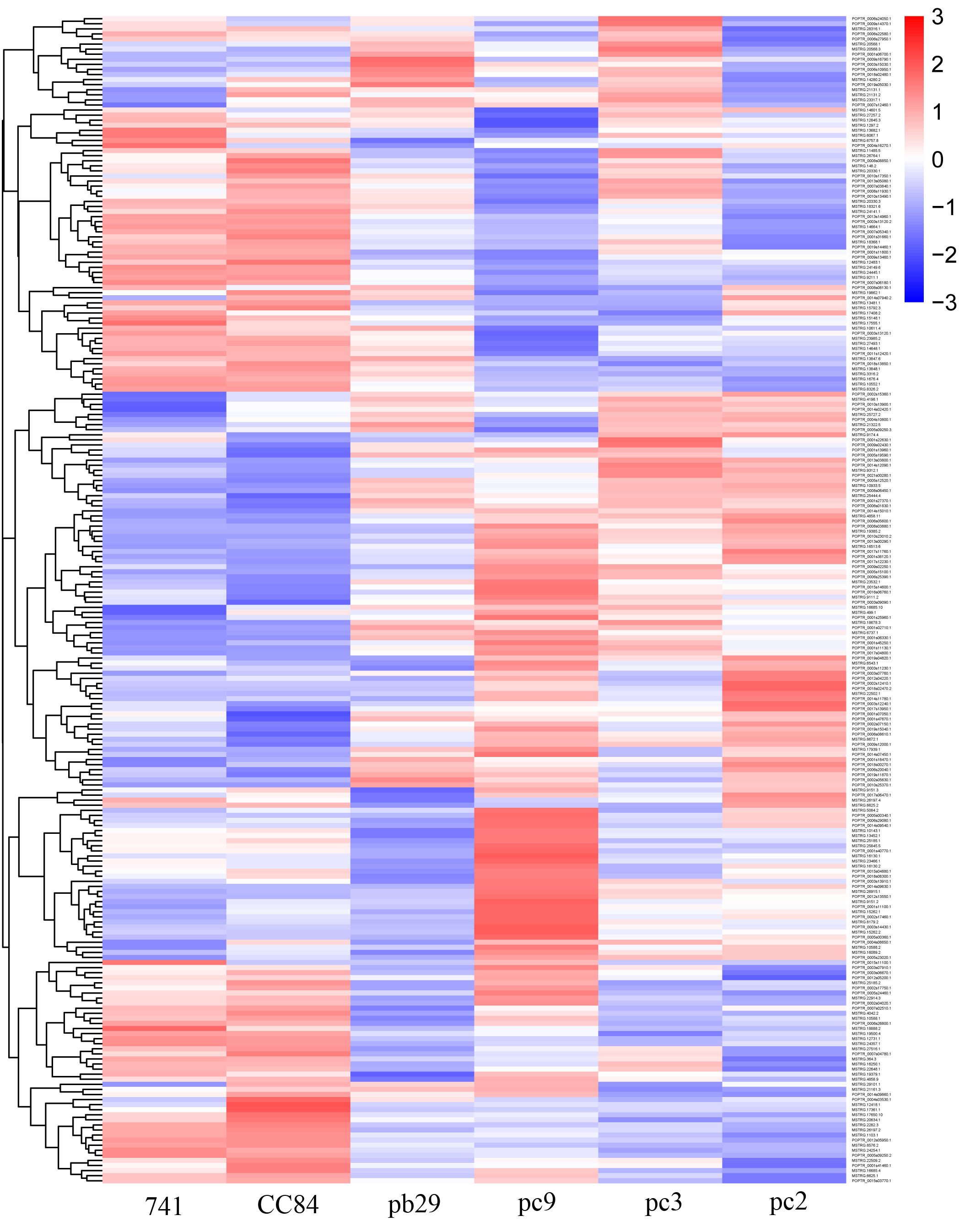


Supplementary Figure S5 The expression levels of all transcription factors


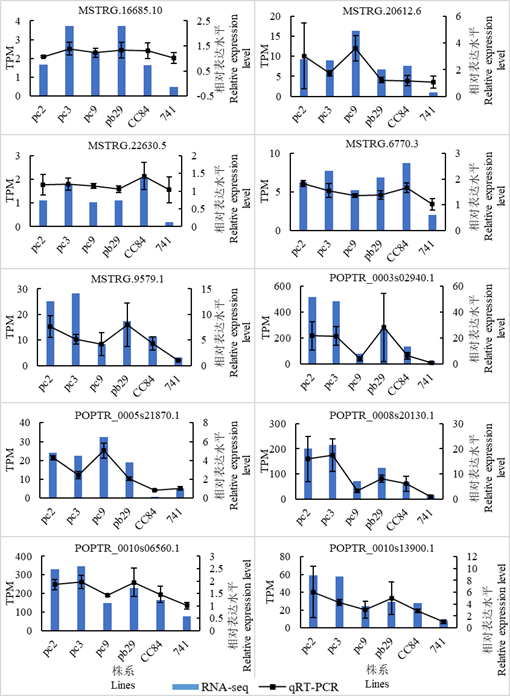


Supplementary Figure S6 Comparison between RT qPCR results and transcriptome sequencing results


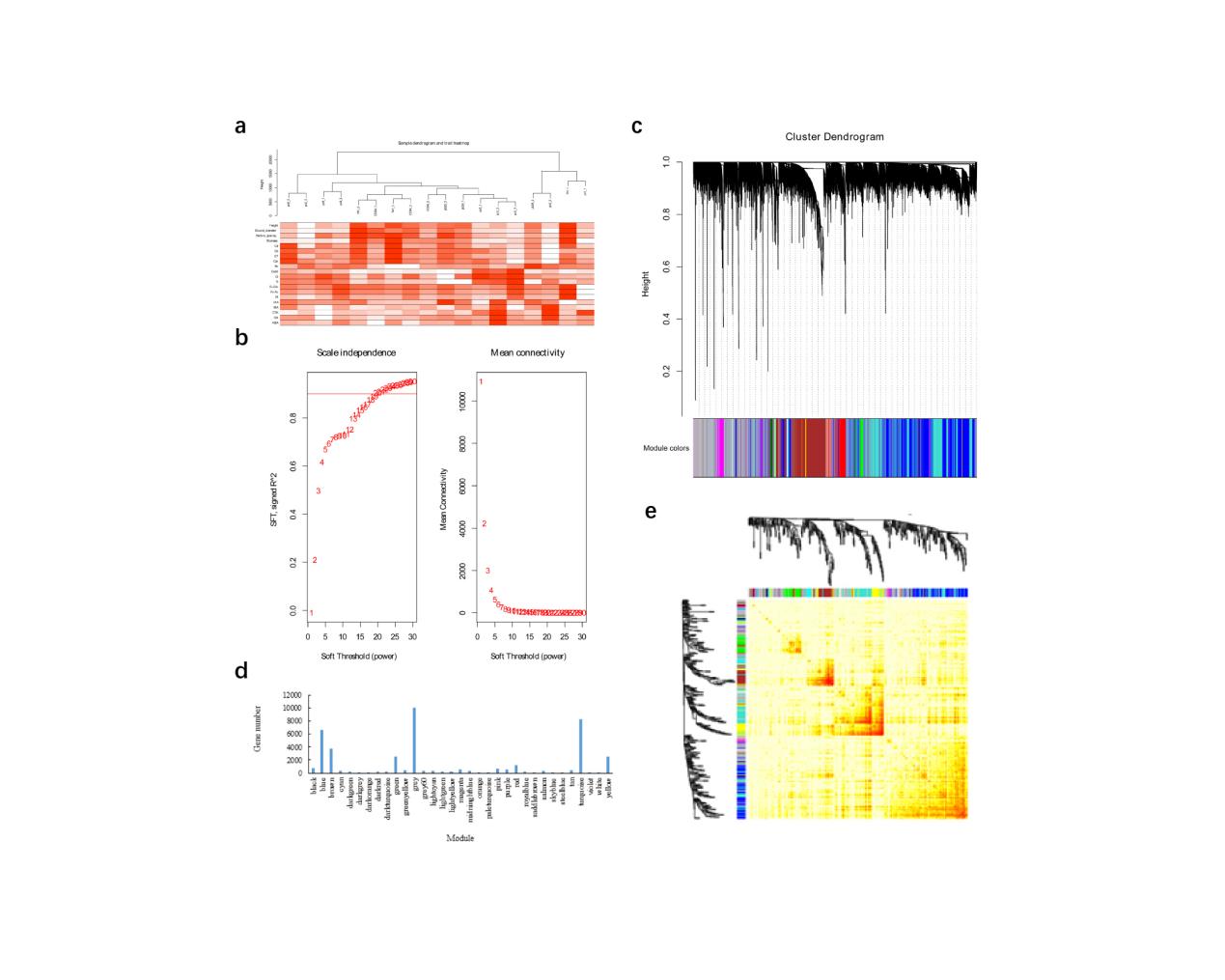


Supplementary Figure S7 The expression data of all genes in the transcriptome were used to generate a sample clustering diagram of gene expression levels (Figure S7a). Based on the screening of weight values, β = 17 was ultimately selected to construct the network (Figure S7b). The dynamic tree cut method was used to merge modules with similar expression patterns, resulting in a total of 33 co-expression modules (Figure S7c). The number of genes in each module was statistically analyzed, with the results shown in Figure S5d.Interactions between modules were visualized using the TOM matrix (Figure S7e).


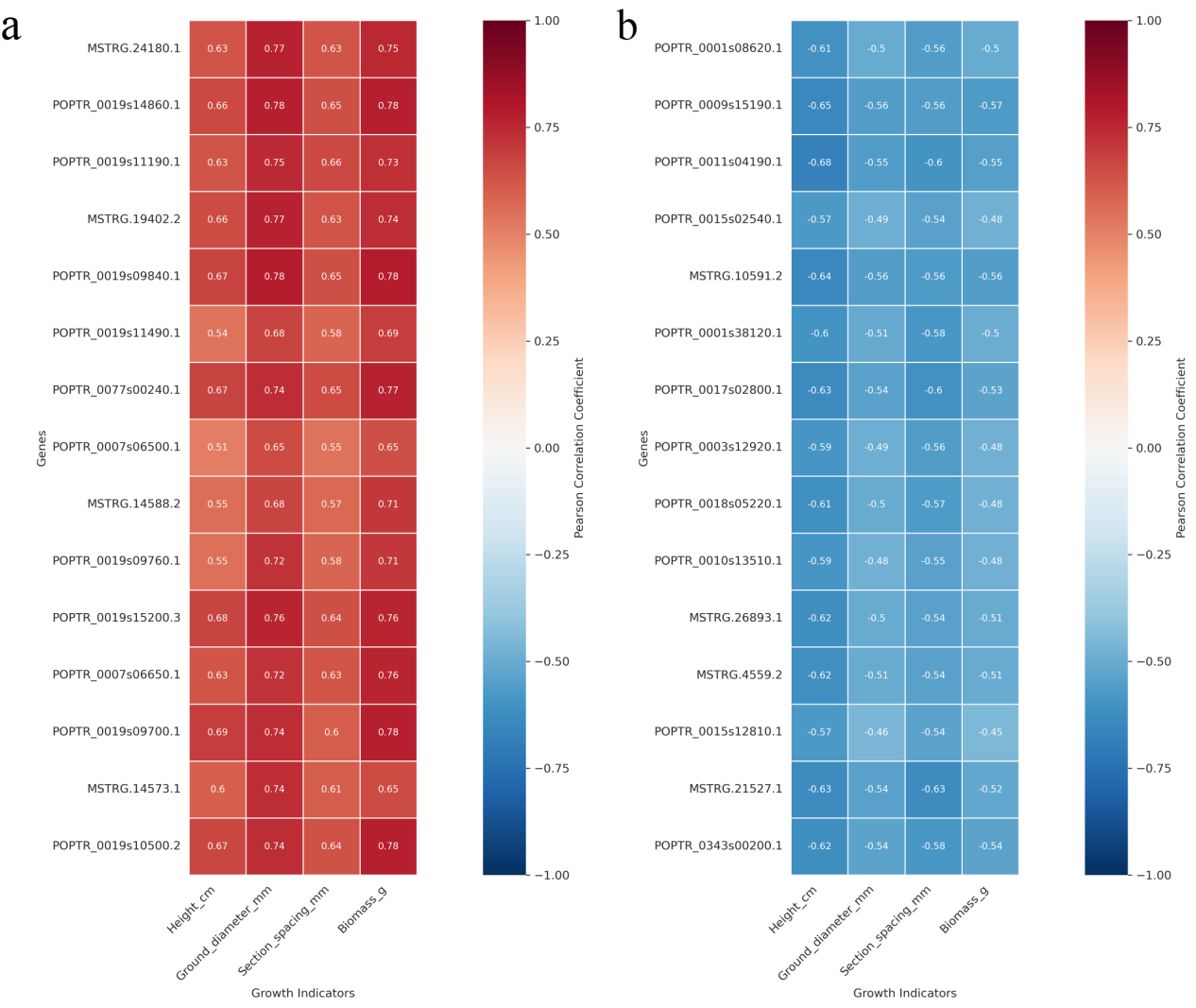


Supplementary Figure S8 Correlations between the expression levels of the top 15 hub genes in the module and four growth traits (plant height, ground diameter, internode length, and biomass).

Figure S8a: Correlation between the expression levels of the top 15 hub genes in the blue module and four growth traits. Figure S8b: Correlation between the expression levels of the top 15 hub genes in the brown module and four growth traits.
